# Restoring the tumour mechanophenotype of vocal fold cancer reverts its malignant properties

**DOI:** 10.1038/s41563-025-02473-7

**Published:** 2026-02-20

**Authors:** Jasmin Kaivola, Karolina Punovuori, Megan R. Chastney, Hind Abdo, Gautier Follain, Mathilde Mathieu, Omkar Joshi, Yekaterina A. Miroshnikova, Fabian Krautgasser, Jasmin Di Franco, James R. W. Conway, Sofia Held, Fabien Bertillot, Jaana Hagström, Antti Mäkitie, Heikki Irjala, Sami Ventelä, Hellyeh Hamidi, Giorgio Scita, Roberto Cerbino, Sara A. Wickström, Johanna Ivaska

**Affiliations:** 1 Turku Bioscience Centre, University of Turku and Åbo Akademi University, Turku, Finland; 2 Stem Cells and Metabolism Research Program, Faculty of Medicine, University of Helsinki, Helsinki, Finland; 3 IFOM ETS—The AIRC Institute of Molecular Oncology, Milan, Italy; 4 Faculty of Science and Engineering, Cell Biology, Åbo Akademi University, Turku, Finland; 5 Turku Collegium for Science, Medicine and Technology (TCSMT), Turku, Finland; 6 Laboratory of Molecular Biology, National Institute of Diabetes and Digestive and Kidney Diseases, National Institutes of Health, Bethesda, MD USA; 7 Faculty of Physics, University of Vienna, Vienna, Austria; 8 Vienna Doctoral School in Physics, University of Vienna, Vienna, Austria; 9 Department of Biochemistry and Developmental Biology, Faculty of Medicine, University of Helsinki, Helsinki, Finland; 10 Department of Cell and Tissue Dynamics, Max Planck Institute for Molecular Biomedicine, Münster, Germany; 11 Department of Oral Pathology and Radiology, Dental Institute, University of Turku and Turku University Hospital, Turku, Finland; 12 Translational Cancer Research Unit, University of Helsinki, Helsinki, Finland; 13 Department of Otorhinolaryngology—Head and Neck Surgery, University of Helsinki and Helsinki University Hospital, Helsinki, Finland; 14 Research Program in Systems Oncology, Faculty of Medicine, University of Helsinki, Helsinki, Finland; 15 Division of Ear, Nose and Throat Diseases, Department of Clinical Sciences, Intervention and Technology, Karolinska Institute and Karolinska University Hospital, Stockholm, Sweden; 16 Department of Otorhinolaryngology—Head and Neck Surgery, University of Turku and Turku University Hospital, Turku, Finland; 17 Department of Oncology and Haemato-Oncology, University of Milan, Milan, Italy; 18 Helsinki Institute of Life Science, Biomedicum Helsinki, University of Helsinki, Helsinki, Finland; 19 Department of Life Technologies, University of Turku, Turku, Finland; 20 InFLAMES Research Flagship Center, University of Turku, Turku, Finland; 21 Foundation for the Finnish Cancer Institute, Helsinki, Finland; 22 Western Finnish Cancer Center (FICAN West), University of Turku, Turku, Finland

**Keywords:** Cell adhesion, Soft materials, Cancer models, Extracellular matrix

## Abstract

Increased extracellular matrix deposition and stiffness promotes solid tumour progression. Yet, the precise mechanotransduction pathways, especially in less-studied mechanically responsive cancers, remain poorly understood. Here we address this gap using patient-derived tumour cells from early (mobile, T1) and advanced (immobile, T3) stages of vocal fold cancer, the most common squamous cell carcinoma severely impacting the voice box. We reveal that vocal fold cancer progression is linked to cell surface receptor heterogeneity, a loss of laminin-binding integrins in cell–cell junctions and a flocking mode of collective cell motility. Mimicking the physiological movement of healthy vocal fold tissue with stretching or vibrations decreases oncogenic β-catenin and Yes-associated protein (YAP) nuclear levels in vocal fold cancer. Multiplex immunohistochemistry of vocal fold cancer tumours shows a correlation between the extracellular matrix composition, nuclear YAP and patient survival, concordant with vocal fold cancer sensitivity to oncogenic YAP-TEAD Hippo pathway inhibitors both in vitro and in vivo. Overall, our findings suggest that vocal fold cancer is a mechanically sensitive malignancy, and that the restoration of tumour mechanophenotype or YAP/TAZ targeting represents a tractable anti-oncogenic therapeutic avenue for vocal fold cancer.

## Abstract (web summary)

Vocal fold cancer tumours lose some of their malignant traits when mechanically stimulated by physiological stretch or vibrations, mimicking the opening and closing of vocal folds and phonation.

## Main

**Fig. 1:**
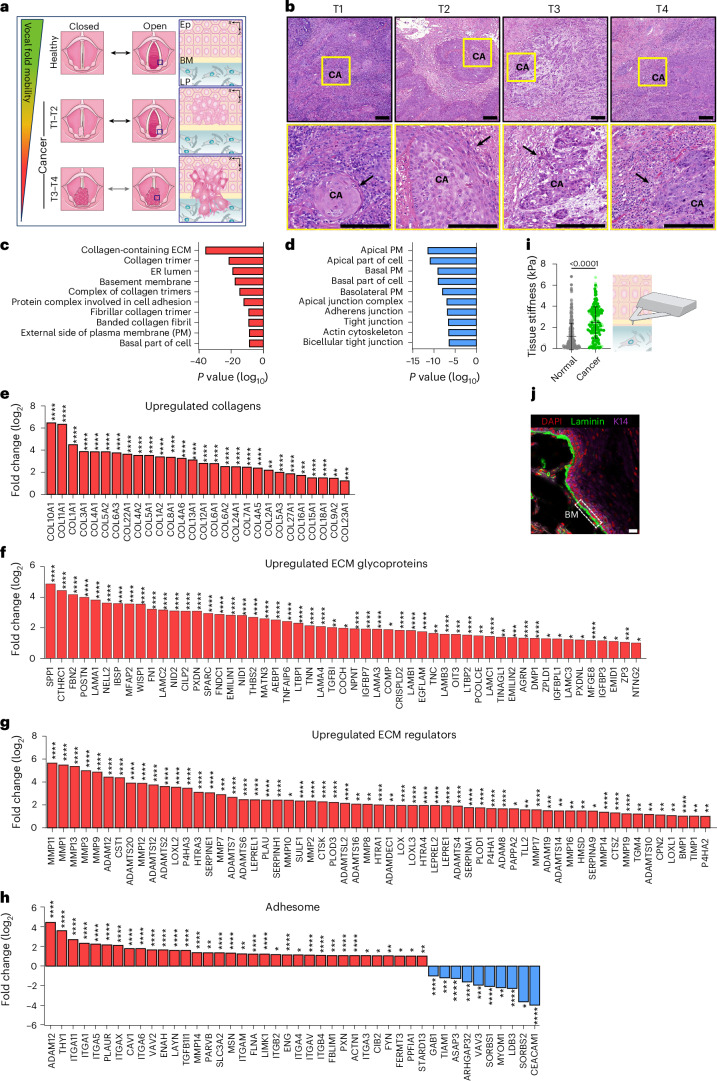
VFC is associated with elevated ECM gene expression and a stiffer tissue. **a**, Schematic of changes in vocal fold mobility and invasion of transformed squamous cells through the basement membrane in VFC progression from T1 to T4. Ep, epithelium; BM, basement membrane; LP, lamina propria. **b**, Haematoxylin and eosin staining of T1–T4 vocal fold squamous cell carcinoma (CA) in patient tissue, with arrows highlighting invasion (representative images taken from a VFC patient dataset). Scale bar, 200 µm. **c**,**d**, Over-represented GO terms in upregulated (**c**) and downregulated (**d**) differentially expressed genes in VFC (T1–T4, *n* = 54) compared with normal (*n* = 12) patient tissue (TCGA data; ROTS; FDR < 0.001). Data represent the mean. **e**–**h**, Differentially upregulated (log_2_[fold change]) collagens (**e**), ECM glycoproteins (**f**), ECM regulators (**g**) and differentially up- and downregulated adhesome^37,86^ genes (**h**) in VFC (T1–T4, *n* = 54) compared with normal (*n* = 12) patient tissue (TCGA data; ROTS; FDR < 0.05) annotated with a Matrisome AnalyzeR^33^. Data represent the mean. **i**, Tissue stiffness (Pa) of normal (*n* = 366 force curves pooled from three biological replicates) and VFC (*n* = 205 force curves pooled from two biological replicates) patient tissue measured by AFM force indentation spectroscopy (mean ± standard deviation (s.d.); each dot represents an AFM cantilever contact point with the specimen; two-tailed Mann–Whitney *U*-test). **j**, Immunofluorescence staining (DAPI, laminin, K14) of normal vocal fold tissue (representative image from one tissue sample is shown). Scale bar, 30 µm. Exact *P* values for **c**–**h** are provided in the Source data. Credit: vocal fold illustrations in **a**, Servier Medical Art (https://smart.servier.com/) under a Creative Commons license CC BY 4.0. Source data

Human vocal folds are composed of three layers (namely, epithelial, basement membrane and lamina propria), with distinct cellular and extracellular matrix (ECM) compositions (Fig. 1a)^1^. Maintaining proper ECM organization is essential for vocal fold epithelium viscoelasticity, as it has been shown that the biomechanical and physiological performance of the vocal folds relies on ECM homeostasis^2,3^. ECM alterations are also linked to numerous pathological conditions, such as cancer^4^. Vocal fold cancer (VFC) remains a major clinical challenge with limited targeted therapy options, and a 34% 5-year survival rate for advanced T3–T4 disease. VFC arises in the stratified squamous epithelium, and as it progresses, the squamous cells in the epithelial layer breach the underlying basement membrane, invade into the collagen-rich lamina propria and further into the muscle, leading to mechanical fixation^4–7^, which is a characteristic of T3 and T4 disease (Fig. 1a).

In recent years, there has been a growing appreciation of the role of ECM remodelling and increased deposition in cancer pathogenesis across a multitude of cancer types^8,9^, as the ensuing increase in tissue rigidity alters tissue mechanics and drives disease progression^10–12^. Integrins, the main cellular ECM receptors^13^, act as mechanosensors that probe the physical properties of their surroundings and transmit this information through the cytoskeleton into intracellular biochemical signals and transcriptional changes^14–16^. Enhanced tissue rigidity and integrin engagement trigger key oncogenic signals, such as the stabilization and nuclear translocation of the Hippo signalling pathway transcription factors Yes-associated protein (YAP) and TAZ (refs. ^17,18^). YAP/TAZ are upregulated in various cancers and influence tumour initiation, progression and therapeutic resistance^19–21^. Importantly, this signalling is reciprocal; YAP positively controls focal adhesion (FA) assembly^22^, and integrin–ECM adhesion regulates YAP/TAZ in the squamous epithelium^23^. However, it remains unknown whether changes in ECM and cell mechanics play a role in VFC. Further, it is not known whether immobility caused by fixation contributes to VFC malignancy or correlates with patient outcome.

The role of the ECM and mechanical forces in tumour development have predominantly been investigated in solid tumours arising from non-motile tissues, such as the mammary gland, brain and pancreas, with a focus primarily on the outcomes of increased rigidity^24^. Recently, continuous dynamic mechanical challenge to the lung epithelium was shown to increase nuclear YAP in ventilated rat lungs^25^, and cell stretching was shown to trigger changes in the heterochromatin architecture and nuclear softening^26^. Furthermore, in the mechanically active mouse colon, high-frequency pulsatile mechanical stresses maintain the physiological level of stem cells through the mechanosensitive Ret kinase, whereas magnetically generated pressure induces the tumourigenic β-catenin pathway, suggesting distinct responses to pulsatile and static mechanical stresses^27,28^. Due to the unique biomechanical properties of the vocal fold, we sought to understand the role of cell–matrix and cell–cell adhesion, as well as their mechanical regulation in VFC. Given the established role of mechanical stress in tumour progression, we investigated how voice frequency mechanical strains impact tumourous vocal fold tissues, where transition from a freely vibrating to an immobilized state has been shown to correlate with poor prognosis.

## VFC is associated with elevated ECM expression

**Extended Data Fig. 1:**
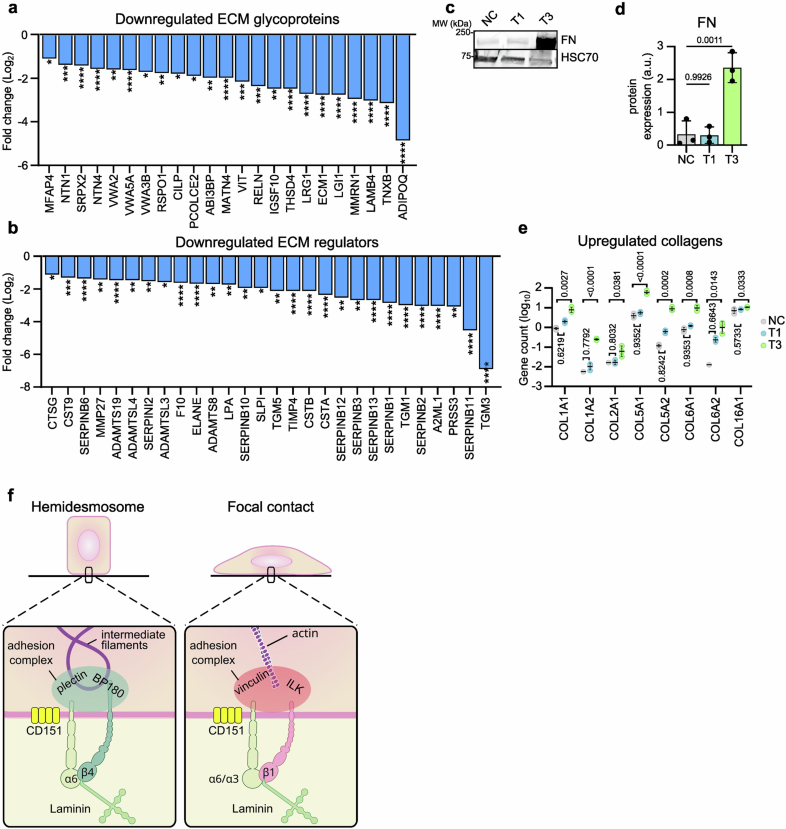
Vocal fold cancer is associated with elevated ECM component gene expression and tissue stiffening. **a** & **b**, Differentially downregulated (fold change, log_2_) ECM glycoproteins (**a**) and ECM regulators (**b**) in VFC tissue (T1-T4, *n* = 54) compared to normal tissue (*n* = 12) in patients (TCGA-data; reproducibility-optimized test statistic (ROTS); false discovery rate (FDR) < 0.05). Data represent the mean. See source data for exact p-values; *<0.05, **<0.01, ***<0.001, ****<0.0001. **c** & **d**, Representative immunoblot (**c**) and quantification (**d**) of fibronectin (FN) protein expression in NC and VFC T1 and T3 cells (three biological replicates). Data are mean ± standard deviation (s.d.). Ordinary one-way ANOVA followed by post hoc Dunnett’s multiple comparisons test was used to assess statistical significance. **e**, Gene count (log_10_; number of reads that map to each gene, RNA-seq-data) of upregulated collagens in NC cells and VFC T1 and T3 cells (three biological replicates). Data are mean dot plots ± s.d. Ordinary one-way ANOVA followed by Dunnett’s multiple comparisons test was used to assess statistical significance. **f**, Schematic of laminin-binding integrins in hemidesmosomes (α6β4) and focal contacts (α6β1 and α3β1) connecting epithelial cells to the keratin cytoskeleton via BP180 and plectin or to the actin cytoskeleton via ILK and vinculin. Related to Fig. 1. Source data

Earlier studies have demonstrated that vocal fold trauma, such as scarring, can lead to fibronectin and collagen accumulation in the tissue^3,29^. Moreover, VFC progression causes vocal fold immobility as the squamous cell carcinoma invades the underlying muscle and tissues of the neck (Fig. 1a,b)^4^. VFC staging is based on the mobility status of the vocal folds and invasion into surrounding tissues; in T1–T2, the vocal folds move normally, whereas in T3–T4, mechanical fixation renders the vocal fold(s) immobile (Fig. 1a,b)^4^. We aimed to investigate the ECM composition and stiffness of VFC compared with normal tissue in patient samples. First, we analysed head and neck cancer RNA-sequencing data generated by The Cancer Genome Atlas (TCGA) research^30^, focusing specifically on samples with patient reports mentioning the involvement of the vocal fold tissue (glottic larynx). Considering the low number of T1 and T2 cancer samples (*n* = 4), we pooled all the cancer samples together. Normal (*n* = 12) and cancer (T1–T4; *n* = 54) samples were compared to determine differentially expressed genes; 2,041 genes were upregulated and 1,629 were downregulated in the cancer samples compared with normal tissue (false-discovery rate, FDR < 0.05). Gene ontology (GO) enrichment analysis^31,32^ revealed an over-representation of ECM and collagen-related GO terms such as collagen-containing ECM, basement membrane and protein complex involved in cell adhesion in the upregulated genes in cancer (Fig. 1c). Conversely, the over-represented GO terms in downregulated genes were linked to cell junctions and apical regions of the cell (Fig. 1d). We further determined the genes encoding ECM and ECM-associated proteins in the dataset using a Matrisome AnalyzeR^33,34^. Strikingly, all differentially expressed collagens were upregulated, including collagens I, III, IV and V that are abundant in the vocal folds^35^ (Fig. 1e). Among the 76 differentially expressed ECM glycoprotein genes, 53 were upregulated and 23 were downregulated (Fig. 1f and Extended Data Fig. 1a). The upregulated genes included fibronectin (*FN1*) and laminin 332 chains (*LAMA3*, *LAMB3* and *LAMC2*), which can function as autocrine tumour promoters in squamous cell carcinoma through laminin-binding integrins α6β4 and α3β1 (ref. ^36^). Moreover, 59 ECM regulator genes were upregulated (Fig. 1g) and 28 were downregulated (Extended Data Fig. 1b). The upregulated lysyl oxidase (*LOX*) and its homologues (*LOXL1*, *LOX2* and *LOXL3*), which covalently crosslink collagens to elastin, and metalloproteinases (*MMP14*, *MMP2*, *MMP10*, *MMP1*, *MMP7*, *MMP19*, *MMP9*, *MMP12*, *MMP11*, *MMP13*, *MMP3*, *MMP17*, *MMP16* and *MMP8*) collectively allude to extensive ECM remodelling and stiffening in cancerous tissue compared with normal tissue. Finally, we investigated the genes encoding integrin adhesome proteins^37^ and observed a significant upregulation of many integrin adhesion complex proteins, including several integrins (Fig. 1h).

## VFC is associated with tissue stiffening

To further investigate the changes in ECM composition on the cellular level, we compared T1 (UT-SCC-11; 58-year-old male) and T3 (UT-SCC-103; 51-year-old male) patient-derived VFC cell lines, generated at the University of Turku^38–40^, to non-cancerous (NC) (HaCaT; immortalized human keratinocytes) cells. Western blot analysis confirmed fibronectin upregulation in T3 cancer cells compared with NC cells and T1 cancer cells (Extended Data Fig. 1c,d). Several collagens were also upregulated in T3 cells compared with NC cells in our RNA-sequencing data (Extended Data Fig. 1e). To investigate if the altered ECM production impacted tissue stiffness, we performed force indentation spectroscopy on NC ‘normal’ (*n* = 3) and cancer (*n* = 2) patient samples (obtained from vocal fold surgery at the Turku University Hospital) using atomic force microscopy (AFM; Fig. 1i). The normal tissue was also stained to visualize the epithelial compartment and lamina propria, clearly separated by an intact basement membrane (Fig. 1j). Measurements of the elastic modulus of these tissues confirmed a 3.2-fold increase in cancer tissue stiffness (2.441 ± 1.479 kPa) compared with normal tissue (0.751 ± 0.341 kPa; Fig. 1i,j). Taken together, these results demonstrate ECM component over-expression and significant tissue stiffening in VFC.

## Altered laminin integrin localization in VFC

**Fig. 2:**
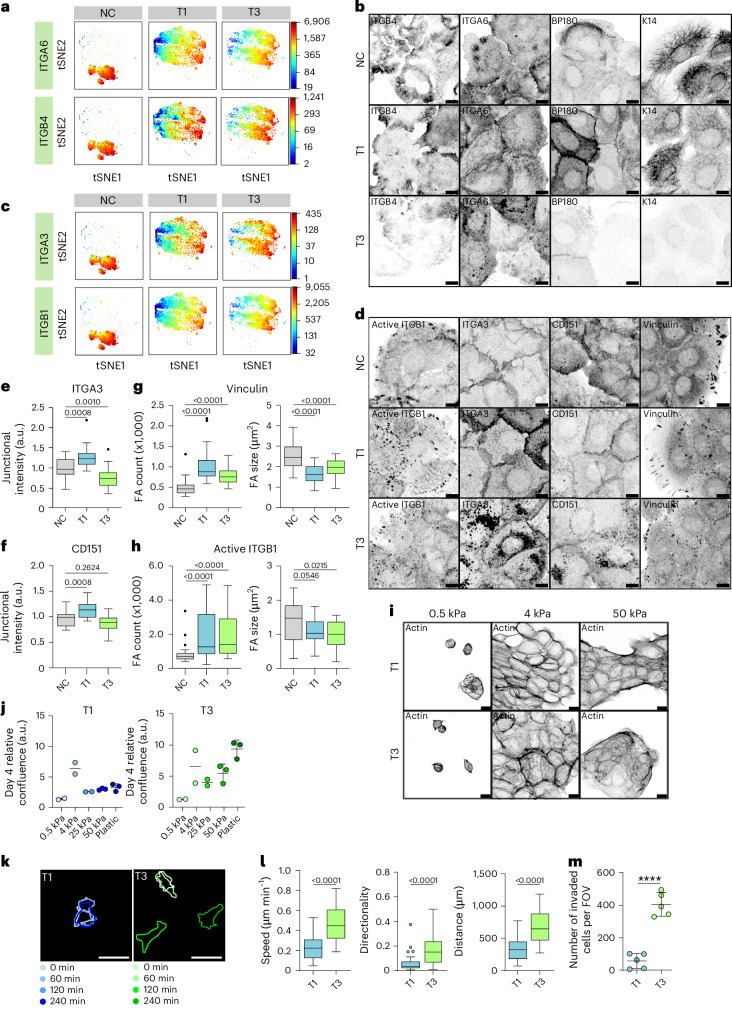
VFC laminin-binding integrin localization, cell proliferation, migration and invasion. **a**, ITGA6 and ITGB4 single-cell surface expression (*t*-distributed stochastic neighbour embedding (*t*-SNE) visualization) in NC and VFC T1 and T3 cells. **b**, Representative confocal immunofluorescence images of NC and VFC T1 and T3 cells stained as indicated (three biological replicates). **c**, ITGA3 and ITGB1 single-cell surface expression as in **a**. **d**, Representative confocal images of NC and VFC T1 and T3 cells stained as indicated (12G10, active ligand-engaged ITGB1 antibody; three biological replicates). **e**,**f**, Quantification of junctional ITGA3 (**e**) and CD151 (**f**) (ITAG3, *n* = 28 (NC and T3) and 27 (T1) FoVs; CD151, *n* = 20 FoVs for all; FoVs pooled from four (**e**) and three (**f**) biological replicates; ordinary one-way ANOVA followed by Holm–Šídák’s multiple comparisons). **g**,**h**, FA number (count) and size based on vinculin (**g**) and active ITGB1 (**h**) staining (NC, vinculin, *n* = 29 (count) and *n* = 30 (size), ITGB1, *n* = 28 (count) and *n* = 30 (size); T1, *n* = 30 for all; T3, vinculin, *n* = 30 (count and size), ITGB1, *n* = 29 (count) and *n* = 30 (size) cells pooled from three biological replicates; ordinary one-way ANOVA followed by Holm–Šídák’s multiple comparisons (FA size) and one-way Kruskal–Wallis followed by Dunn’s multiple comparisons (FA count)). **i**, Representative confocal images of VFC T1 and T3 cells on collagen I- and fibronectin-coated hydrogels stained for actin (three biological replicates). **j**, Relative VFC T1 and T3 cell confluence (day 4 normalized to 0 h) on collagen I- and fibronectin-coated hydrogels and plastic (mean ± s.d.; *n* = 2 (0.5, 4 and 25 kPa) and 3 (50 kPa and plastic) biological replicates). **k**,**l**, Representative outlines (**k**) and quantification (**l**) of single-cell migration on 50-kPa hydrogels (T1, *n* = 34 and T3, *n* = 29 cells pooled from two biological replicates; two-tailed Mann–Whitney test). **m**, T1 and T3 cell invasion (number of invaded cells per FoV) in a Matrigel invasion assay (45 h; mean ± s.d.; *n* = 5 FoVs pooled from two biological replicates). Tukey box plots show the median and interquartile range (IQR). Whiskers extend to 1.5 times the IQR. Scale bars, 10 μm (**b** and **d**); 20 μm (**i**); 50 μm (**m**). Source data

**Extended Data Fig. 2:**
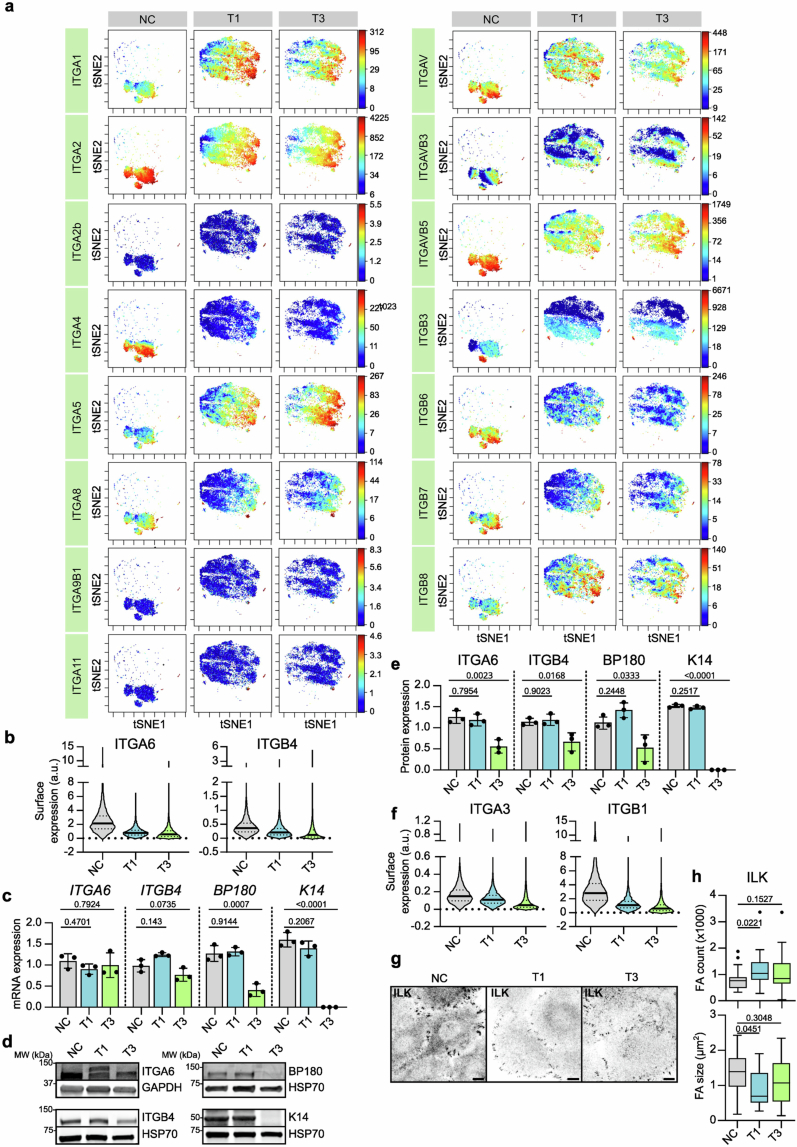
Expression and subcellular localization of laminin-binding integrins is altered in vocal fold cancer. **a**, t-SNE visualization of ITGA1, ITGA2, ITGA2b, ITGA4, ITGA5, ITGA8, ITGA9B1, ITGA11, ITGAV, ITGAVB3, ITGAVB5, ITGB3, ITGB6, ITGB7 and ITGB8 single-cell surface expression (MassCytof) in NC cells and vocal fold T1 and T3 cancer cells. **b**, Violin plot representation of mean ITGA6 and ITGB4 single-cell surface expression (MassCytof) in NC cells and vocal fold T1 and T3 cancer cells. **c**, Relative ITGA6, ITGB4, BP180 and K14 mRNA expression levels (gene count; number of reads that map to each gene, RNA-seq-data) in NC cells and vocal fold T1 and T3 cancer cells (three biological replicates). Data are mean ± s.d. Ordinary one-way ANOVA followed by Dunnett’s multiple comparisons test was used to assess statistical significance. **d** & **e**, Representative immunoblots (**d**) and quantification (**e**) of relative ITGA6, ITGB4, BP180 and K14 protein expression levels in NC cells and vocal fold T1 and T3 cancer cells (three biological replicates). Data are mean ± s.d. Ordinary one-way ANOVA followed by Dunnett’s multiple comparisons test was used to assess statistical significance. **f**, Violin plot representation of mean ITGA3 and ITGB1 single-cell surface expression (MassCytof) in NC and VFC T1 and T3 cells. **g**, Representative ILK confocal immunofluorescence images of NC cells and VFC T1 and T3 cells (three biological replicates). Scale bar 10 μm. **h**, Quantification of FA number (count) (left) and size (right) using ILK as a marker in NC, T1 and T3 cells (NC, *n* = 19 (FA count) and 21 (FA size); T1, *n* = 20 (FA count) and 21 (FA size); T3, *n* = 19 (FA count) and 21 (FA size); pooled from three biological replicates. Kruskal-Wallis test followed by post hoc Dunn’s multiple comparisons test was used to assess statistical significance. Box plots are Tukey box plots and show the median and interquartile range (IQR) and whiskers extend to 1.5 times the IQR. Related to Fig. 2. Source data

The patient data indicated an increase in laminin-binding integrins α3, α6 and β4. The α6β4-integrin heterodimer is found in hemidesmosomes, whereas integrins α3 and α6 form dimers with integrin β1 in focal contacts^41,42^ (Extended Data Fig. 1f). To determine whether these changes were recapitulated in the patient-derived cell lines, we used mass cytometry for a high-dimensional phenotypic analysis of the cell surface expression of 42 adhesion and signalling receptors, including 19 integrins, on a single-cell level. The NCs had largely homogeneous expression profiles, whereas the cancer cell lines showed a high degree of variation (Extended Data Fig. 2a). The integrins α6 and β4 cell surface expression levels were heterogeneous, ranging from high to very low in cancer cells compared with NC cells based on mass cytometry analysis (Fig. 2a and Extended Data Fig. 2b) and confocal immunofluorescence imaging (Fig. 2b). Staining of α6β4-associated hemidesmosome components BP180 (collagen XVII) and keratin 14 (K14) reflected a similar heterogeneity and indicated a clear overall loss of hemidesmosomes and their associated intermediate filament cytoskeleton in T3 cancer cells. Similar changes were also detected on bulk mRNA and protein levels for α6, β4, BP180 and K14 (Extended Data Fig. 2c–e). Cell surface expression of integrins α3 and β1 was also heterogeneous in cancer cells (Fig. 2c and Extended Data Fig. 2f), and confocal immunofluorescence imaging demonstrated that this was linked to a striking difference in subcellular integrin localization, rather than absolute changes in protein expression (Fig. 2d). Integrin α3 unexpectedly localized predominantly to cell–cell junctions in NC and T1 cells, whereas junctional localization was significantly decreased, and shifted to endosome-like intracellular structures in T3 cancer cells (Fig. 2d,e). The same trend was evident for the tetraspanin CD151, which interacts with α3β1 integrin with high affinity, localizing to focal contacts and hemidesmosomes^43^ (Fig. 2d,f). Furthermore, the cancer cells had an increased number of smaller vinculin-positive, active integrin β1 (12G10 antibody)-positive and integrin-linked kinase (ILK)-positive cell–matrix adhesions, compared with NC cells (Fig. 2g,h and Extended Data Fig. 2g,h). Intriguingly, in addition to junctional localization, integrin α3 also localized to cryptic lamellipodia, which regulate epithelial cell migration^44^, in NC and T1 cells (Fig. 2d). These marked changes in laminin-binding integrins imply that cell–cell and cell–matrix adhesions are altered in VFC.

## ECM stiffness supports VFC cell proliferation

**Extended Data Fig. 3:**
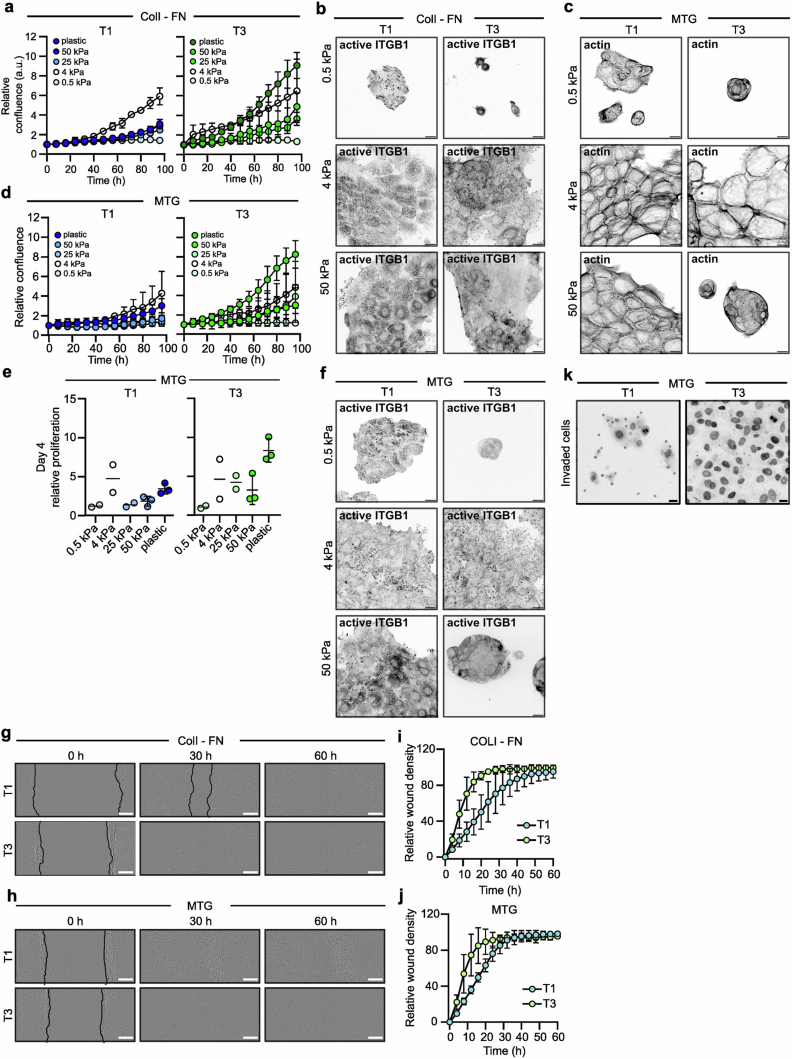
Stiffening of vocal fold tissue supports increased cell proliferation, migration and invasion. **a**, Proliferation of T1 and T3 VFC cells on hydrogels of varying stiffnesses (0.5 kPa, 4 kPa, 25 kPa, 50 kPa) and plastic coated with collagen I and fibronectin (ColI-FN) (mean ± s.d.; *n* = 2 (0.5 kPa, 4 kPa, 25 kPa) *n* = 3 (50 kPa; plastic) biological replicates). **b**, Representative active ITGB1 (12G10) confocal immunofluorescence images of T1 and T3 VFC cells on 0.5, 4 or 50 kPa hydrogels coated with ColI-FN (three biological replicates). Scale bar 20 μm. **c**, Representative actin confocal immunofluorescence images of T1 and T3 VFC cells on 0.5, 4 and 50 kPa hydrogels coated with Matrigel (three biological replicates). Scale bar 20 μm. **d, e**, Proliferation of T1 and T3 VFC cells (**d**) on hydrogels of varying stiffness and plastic coated with Matrigel and confluence at end-point (**e**) (mean ± s.d.; *n* = 2 (0.5 kPa, 4 kPa, 25 kPa) *n* = 3 (50 kPa, plastic) biological replicates). **f**, Representative active ITGB1 (12G10) confocal immunofluorescence images of T1 and T3 VFC cells on 0.5, 4 or 50 kPa hydrogels coated with Matrigel (*n* = 3 biological replicates). Scale bar 20 μm. **g** & **h**, Representative phase-contrast images of T1 and T3 VFC cells wound healing assay at different timepoints (0, 30 and 60 h) on ColI-FN (**g**) or Matrigel (**h**) coated plates. Scale bar 20 μm (*n* = 3). **i** & **j**, Relative wound density of T1 and T3 VFC cells on collagen I and fibronectin (**i**) or Matrigel (**j**) coated plates (*n* = 3). Data are mean ± s.d.; Statistical significance was assessed using Kruskal-Wallis test followed by post hoc Dunn’s multiple comparisons test. Related to Fig. 2. Source data

As we detected an increase in patient tissue stiffness and ECM expression in cancer, we set out to determine whether changes in stiffness influence VFC cell proliferation. We monitored cell proliferation over 4 days on plastic and hydrogels of different stiffness (0.5 kPa, 4 kPa, 25 kPa and 50 kPa) coated with collagen I and fibronectin or Matrigel (mainly composed of laminin and collagen IV). Both T1 and T3 cells proliferated slowly on 0.5 kPa, with T3 cells (and to a lesser degree, T1 cells), demonstrating increased proliferation on plastic. In particular, the highest proliferation occurred with T1 cells on the 4-kPa substrate—closely matching the stiffness of native VFC tissue in vivo (Fig. 2i,j, Extended Data Fig. 3a and Supplementary Videos 1–6). We detected more active β1 integrin in adhesions in T1 and T3 cells compared with NCs and better cell spreading on stiff compared with soft (Fig. 2h–j and Extended Data Fig. 3b). Similar data to the collagen I and fibronectin conditions were obtained on Matrigel-coated plastic and hydrogels (Extended Data Fig. 3c–f and Supplementary Videos 7–12).

## ECM stiffness supports VFC cell migration

As single cells, T3 cells demonstrated increased speed, accumulated distance and directionality compared with T1 cells on collagen I- and fibronectin-coated 50-kPa hydrogels (Fig. 2k,l). Moreover, T3 collective cell migration (as a sheet in wound-healing experiments) was significantly faster compared with T1 cells both on collagen I- and fibronectin-coated and Matrigel-coated plastic plates (Extended Data Fig. 3g–j). Accordingly, T3 cells invaded effectively through Matrigel transwell inserts (45 h), whereas only a small number of T1 cells were able to invade (Fig. 2m and Extended Data Fig. 3k). Taken together, these data indicate VFC cell proliferation and migration are positively regulated by increased ECM rigidity.

## Laminin-binding integrins modulate monolayer dynamics

**Extended Data Fig. 4:**
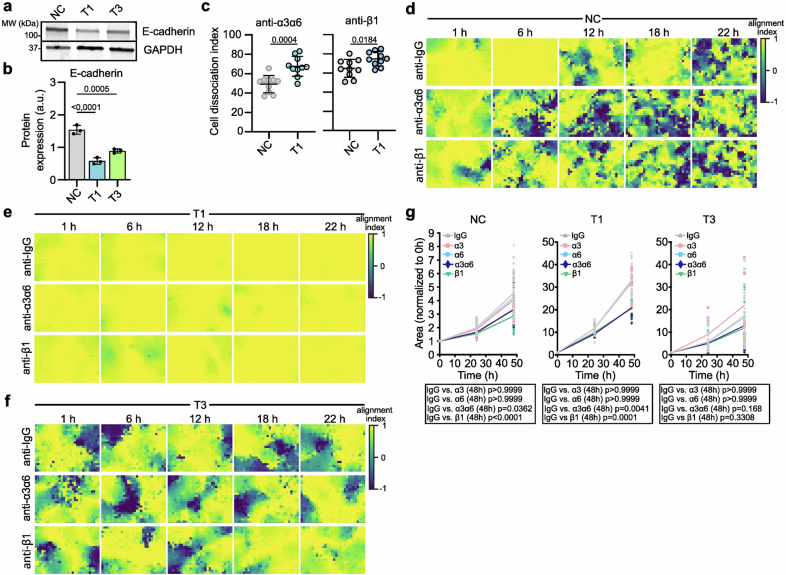
Inhibition of laminin-binding integrins modulates monolayer dynamics and disrupts cell clustering in 3D-spheroids. **a** & **b**, Representative immunoblot (**a**) and quantification (**b**) of E-cadherin protein expression in NC and VFC T1 and T3 cells (mean ± s.d., three biological replicates). **c**, Quantification of the cell dissociation index for NC and VFC T1 cells treated with anti-α3α6 or anti-β1 integrin blocking antibodies (mean ± s.d., NC, *n* = 10 (anti-α3α6) and 10 (anti-β1); T1 *n* = 10 (anti-α3α6) and 10 (anti-β1); pooled from three biological replicates). **d-f**, Graphic visualization of the cell velocity alignment index in NC (**d**), VFC T1 (**e**) and T3 (**f**) cells undergoing jamming and treated with anti-α3α6 or anti-β1 integrin blocking antibodies. **g**, Quantification of normalized wetting area of NC and VFC T1 and T3 spheroids treated with anti-α3α6 or anti-β1 integrin blocking antibodies (*n* = 3). The time evolution of the normalized area is plotted. IgG data are also shown in Fig. 3k comparing NC and T1 and T3 spheroid area. Data are mean ± s.d., Statistical significance was assessed using the Kruskal-Wallis test followed by post hoc Dunn’s multiple comparisons test. Related to Fig. 3. Source data

The α3β1-integrin cell–cell junction localization in normal squamous cells was reported more than two decades ago^45^. Although the role of the α3β1-integrin receptor in mediating cell–matrix adhesion and controlling cell polarity in stratified epithelia is well established both in vitro and in vivo^46^, its role in squamous epithelial cell intercellular adhesion has been controversial and the molecular details remain elusive^47^. To explore the functional role of laminin-binding β1 integrins in VFC, we treated cells with integrin α3-blocking (P1B5), α6-blocking (P5G10) and β1-blocking (mAb13) antibodies. Live-cell imaging of sparse cell clusters revealed the retraction of junctional and cell-edge lamellipodia with a concomitant slowing of cell movement in all cell lines, particularly in NC cells, after the dual inhibition of integrins α3 and α6 (Supplementary Videos 13–15). All of the cell types expressed E-cadherin, with significantly lower expression in cancer cells compared with NC cells based on western blot analysis (Extended Data Fig. 4a,b). Blocking E-cadherin had the opposite effect to integrin inhibition; the weakened cell–cell adhesions supported cell colony scattering by reducing cell–cell coordination and increasing cell elongation and movement (Supplementary Videos 16–18).

## Laminin-binding integrins regulate spheroid compaction

**Fig. 3:**
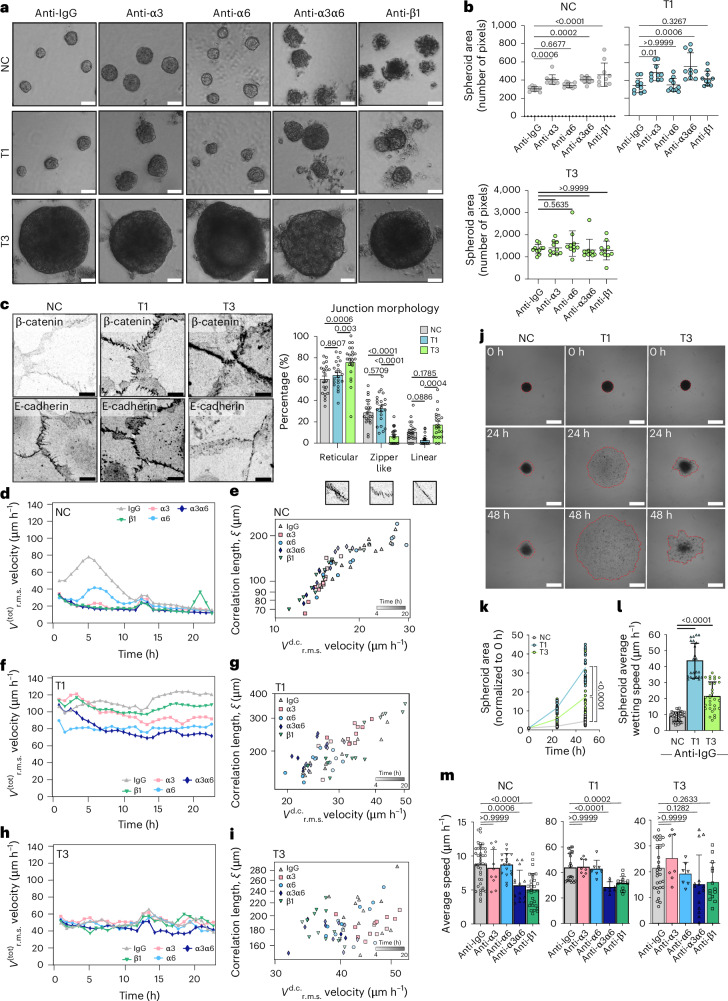
Inhibiting laminin-binding integrins affects monolayer dynamics and cell clustering. **a**,**b**, Representative phase contrast images (**a**) and quantification (**b**) of NC and VFC T1 and T3 spheroid size in 3D Matrigel cultures treated with IgG control or integrin-blocking antibodies (anti-α3, anti-α6, anti-α3α6 and anti-β1) for 11 days (NC; *n* = 9 (IgG) and n = 10 for all other conditions; T1 and T3, *n* = 10 for all conditions; average spheroid size per FoV pooled from three biological replicates; data are mean ± s.d.; Kruskal–Wallis test followed by Dunn’s multiple comparisons test). Scale bar, 50 μm. **c**, Representative β-catenin and E-cadherin confocal immunofluorescence images and the quantification of junction morphology of NC and VFC T1 and T3 cells (NC, *n* = 22; T1, *n* = 22 and T3, *n* = 22; pooled from three biological replicates). Data are mean ± standard error of the mean (s.e.m.). Two-way ANOVA followed by Tukey’s multiple comparisons test was used to assess the statistical significance. Scale bar, 10 μm. **d**–**i**, Quantification of the total r.m.s. velocity and velocity correlation length of NC (**d** and **e**) and VFC T1 (**f** and **g**) and T3 (**h** and **i**) cells treated with IgG control or integrin-blocking antibodies (anti-α3, anti-α6, anti-α3α6 and anti-β1) for 24 h. **j**–**l**, Representative phase contrast images (**j**), quantification of normalized area (**k**; see also Extended Data Fig. 4g) and average wetting speed (μm h^−1^; **l**) of NC and VFC T1 and T3 spheroids treated with anti-IgG. The contour of the spreading spheroids is outlined in red. **m**, NC, VFC T1 and T3 spheroids treated with IgG control (data shown in **l**) or integrin-blocking antibodies (anti-α3, anti-α6, anti-α3α6 and anti-β1) undergoing wetting. The average speed of active wetting is plotted (mean ± s.d.; three biological replicates; in **l** and **m**, ordinary one-way ANOVA followed by Bonferroni’s multiple comparisons is used). Source data

In a three-dimensional (3D) spheroid model, blocking the subunits of laminin-binding integrins—the common β1 subunit or α3 alone or in combination with integrin α6—resulted in increased spheroid area primarily in NC and T1 cancer cells compared with IgG control (Fig. 3a,b). The observed increase in size was due to reduced spheroid compaction and significantly more dissociated cells (Extended Data Fig. 4c). These data imply a functional role for integrins in NC and T1 cell–cell junctions (Fig. 2b,d). The T3 spheroids grew rapidly into large spheroids and integrin inhibition did not trigger marked spheroid dissociation, concordant with intracellularly localized integrins (Fig. 3a,b).

These data prompted us to investigate VFC cell–cell junctions in more detail. T3 cells exhibited linear junctions (E-cadherin and β-catenin immunofluorescence staining), indicative of lower contractile forces acting on the adhesions, whereas NC and T1 cells had protrusive zipper-like junctions, indicative of higher contractile forces acting on the adhesions (Fig. 3c). To quantitatively capture these differences, we divided junctions into three categories (linear, reticular and zipper like) based on the morphology. In particular, although reticular adhesions were a prominent feature in all cells, there was a near absence of zipper-like junctions and a larger proportion of linear junctions in T3 cells compared with NC cells and T1 VFC cells (Fig. 3c). Overall, these data indicate that the cell–cell junction morphology is altered in VFC cell lines and that integrins contribute to junctional dynamics in NC and T1 VFC.

## VFC cells exhibit a solid-like flocking state

Cell–cell and cell–matrix adhesions are critical determinants of the mechanics and dynamics of multicellular, normal and tumourigenic cell assemblies. At a critical cell density, normal epithelia cease to move and cells undergo a jamming phase transition, which is considered a tumour-suppressive mechanism^48,49^. By contrast, phase transition through unjamming and flocking motion, in turn, has been shown to promote collective cancer invasion^50–52^. Thus, we next investigated NC and VFC monolayer dynamics and the impact of integrin inhibition. Particle image velocimetry (PIV; Methods) analysis revealed that untreated NC cells exhibit a progressive reduction in cell motility, quantified by the root mean square (r.m.s.) velocity ${{\boldsymbol{v}}}_{{\mathrm{RMS}}}^{{\mathrm{tot}}}$ (Fig. 3d). We also characterized the jamming transition by extracting the velocity correlation length $\xi$ (expressing the size of a cluster of cells moving together), as well as the drift-corrected total r.m.s. velocity ${{\boldsymbol{v}}}_{{\rm{RMS}}}^{{\rm{d}}{{.}}{\rm{c}}.}({{t}})$ (Fig. 3e and Supplementary Video 19), used to isolate the disordered velocity component, minimizing the effects of drifts. NC monolayers behaved as expected for all treatments, that is, initially large $\xi$ and r.m.s. velocities that simultaneously decreased over time across the jamming transition^53,54^. Inhibiting α3 (P1B5), α6 (P5G10) and β1 (mAb13) integrins significantly and robustly reduced the collective motion, resulting in an accelerated transition towards a jammed state, characterized by a progressive loss of degree of alignment in the cell velocity (Extended Data Fig. 4d and Supplementary Videos 20–23).

Similar analyses were conducted on T1 and T3 cells (Fig. 3f–i and Supplementary Videos 24–33). In both cases, the total r.m.s. velocity (Fig. 3f,h and Supplementary Videos 24 and 29) remained constant in time, with values consistently larger than the final velocity for the NC cells. For T1 cells, the inhibition of integrin α6 or integrins α3 and α6 together were the most efficient in reducing the total r.m.s. velocity, suggesting a relevant role for these integrins in collective cell motility. By contrast, T3 cell motility was insensitive to integrin inhibition. Plotting the velocity correlation length $\xi$ versus the drift-corrected total r.m.s. velocity ${{\boldsymbol{v}}}_{{\rm{RMS}}}^{{\rm{d}}{{.}}{\rm{c}}.}({\boldsymbol{t}})$ (Fig. 3g,i and Supplementary Videos 25–28,30–33) revealed a complete loss of correlation for T3 cells and an intermediate behaviour for T1 cells, suggesting that in both cases, the tissues are far from a dynamically arrested, jammed state. Consistently, T1 VFC cells displayed cohesive and coordinated movement like bird flocking, with aligned cell velocities spanning the entire field of view (FoV; Extended Data Fig. 4e). Interestingly, these cells maintain long-range coordinated motion even when exposed to anti-integrin treatments. Similar flocking behaviour was detected in T3 cells, although to a lesser extent (Extended Data Fig. 4f). The absence of mutual cell rearrangements in VFC collective motility points to a flocking solid-phase transition mode, characterized by long-range coordinated motility without local cell rearrangements. Interestingly, this state, originally predicted by numerical simulation^55,56^, has only very recently been observed in experiments with NC epithelial cells^57^. Collectively, our data suggest that VFC cells exploit a solid flocking state to enhance long-distance collective motion, possibly contributing to invasion and metastasis in the cancer setting^58^. However, the possible generic role of this novel flocking solid state for cancer remains to be explored in future studies.

## VFC spheroids show faster wetting

In keeping with this finding, we directly tested the ability of NC and VFC 3D spheroids to spread and diffuse onto an ECM-coated substrate by undergoing a ‘wetting’ transition^59–61^. This assay is thought to mimic the early step of local dissemination and depends on both cohesive tensional state and viscoelastic properties of the cell aggregates and the cell–ECM interactions. Both T1 and T3 spheroids on fibronectin-coated plates displayed a significant increase in wetting velocity compared with NC, but with a notable difference in morphodynamics. T1 spheroids rapidly wetted the surface, spreading with an elevated and uniform radial velocity consistent with the flocking solid mode of motion and elevated velocity correlation length $\xi$ of the monolayer motility (Fig. 3j–l). T3 spheroids, however, wetted the surface by extending irregular fronts, with protruding clusters and apparently contractile local regions (Fig. 3j and Supplementary Video 34), consistent with their high contractility and the reduced velocity correlation length $\xi$ of monolayer motility. In NC spheroids, the inhibition of integrin β1 (mAb13) or the simultaneous inhibition of α3 (P1B5) and α6 (P5G10) resulted in a marked reduction in wetting velocity. By contrast, the same integrin perturbations caused a more modest decrease in wetting velocity in T1 spheroids and had no observable effect on T3 spheroids (Fig. 3m and Extended Data Fig. 4g). These results indicate that VFC wetting—particularly in T3 spheroids—is largely independent of cell–matrix adhesion receptors. Instead, the wetting behaviour appears to be primarily governed by the bulk mechanical properties of the 3D spheroids, consistent with the observed patterns of collective flocking dynamics.

## Mechanical stimuli induce VFC cell extrusion

**Fig. 4:**
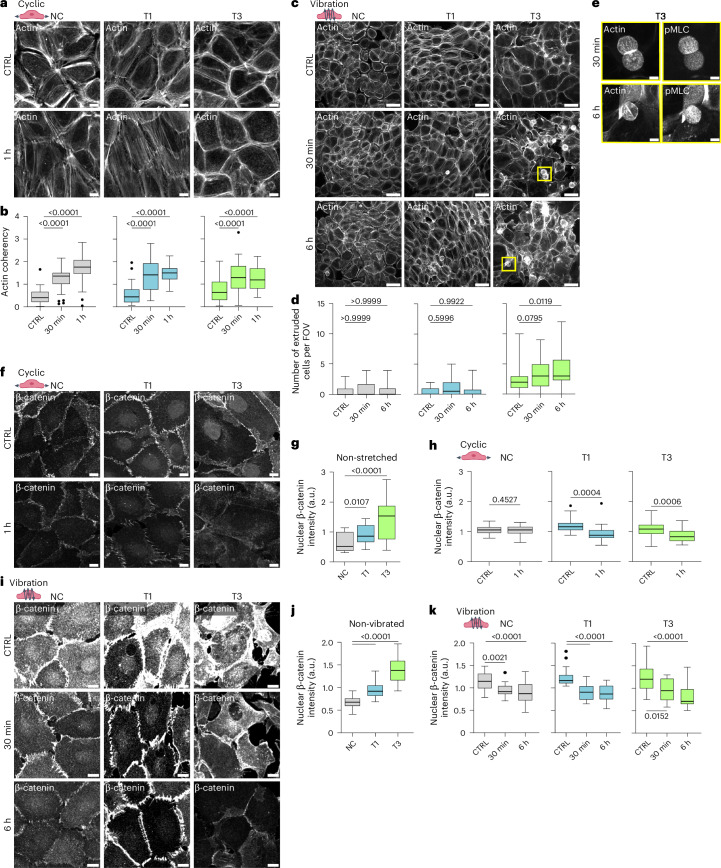
Mechanical stimuli induce cytoskeletal and junctional alterations and cell extrusion. **a**, Representative actin confocal immunofluorescence images in NC and VFC T1 and T3 cells ± stretch (three biological replicates). Scale bar, 10 µm. **b**, Quantification of actin coherency (normalized to the average within biological replicate) in NC and VFC T1 and T3 cells ± stretch (*n* = 48 (coherency per FoV) for each cell line pooled from three biological replicates; Kruskal–Wallis test followed by Dunn’s multiple comparisons). **c**,**d**, Representative actin confocal immunofluorescence images (**c**) and the quantification of extruded cells (**d**) in NC and VFC T1 and T3 cell monolayers ± vibration. **e**, Representative zoomed-in (from the cells in **d**) actin and phospho-myosin light chain 2 (pMLC) confocal immunofluorescence images of extruded T3 VFC cells ± vibration (*n* = 24 for each cell line, pooled from three biological replicates). Scale bar, 50 µm (**c**); 10 µm (**e**). **f**–**h**, Representative β-catenin confocal immunofluorescence images (three biological replicates; **f**) and the quantification of nuclear expression (integrated density per number of nuclei in FoV) in NC and VFC T1 and T3 cells in non-stretched conditions (**g**) and in each cell line ± stretch (**h**; *n* = 26 for each cell line, pooled from three biological replicates; one-way Kruskal–Wallis and Dunn’s post hoc (**g**); paired two-tailed *t*-test (**h**)). Scale bar, 20 µm. **i**–**k**, Representative β-catenin confocal immunofluorescence images (three biological replicates; **i**) and the quantification of nuclear expression (analysed as in **f**) in NC and VFC T1 and T3 cells in non-vibrated conditions (**j**) and in each cell line ± vibration (**k**; *n* = 24 for each cell line, pooled from three biological replicates; Kruskal–Wallis test followed by Dunn’s multiple comparisons). Scale bar, 20 µm. Tukey box plots show the median and IQR. Whiskers extend to 1.5 times the IQR. Source data

**Extended Data Fig. 5:**
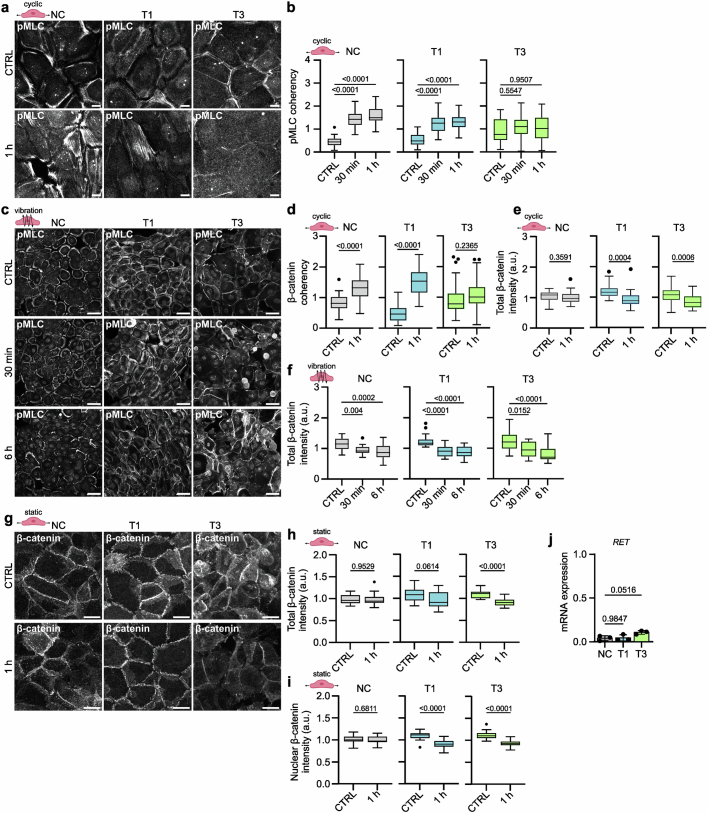
Mechanical stimuli induce cytoskeletal and junctional alterations and cell extrusion in VFC. **a**, Representative phospho-Myosin light chain (pMLC) confocal immunofluorescence images of NC and VFC T1 and T3 cells subjected to cyclic stretch (three biological replicates). **b**, Quantification of pMLC coherency (alignment) of NC and VFC T1 and T3 cells subjected to cyclic stretch (NC *n* = 28, T1 *n* = 28 and T3 *n* = 28; pooled from three biological replicates). Kruskal-Wallis test followed by post hoc Dunn’s multiple comparisons test was used to assess statistical significance. **c**, Representative pMLC confocal immunofluorescence images of NC and VFC T1 and T3 cells subjected to vibration (three biological replicates). Scale bar 20 µm. **d**, Quantification of β-catenin coherency (alignment) in NC and VFC T1 and T3 cells subjected to stretching (NC *n* = 24, T1 *n* = 24 and T3 *n* = 24; pooled from three biological replicates. Paired t-test was used to assess statistical significance). **e** & **f**, Quantification of total β-catenin expression (integrated density per cell number in the field of view (FoV)) for NC and VFC T1 and T3 cells subjected to stretch (**e**) (NC *n* = 24, T1 *n* = 24 and T3 *n* = 24; pooled from three biological replicates) or vibration (**f**) (NC *n* = 24, T1 *n* = 24 and T3 *n* = 24; pooled from three biological replicates. Two-tailed paired t-test (**e**); Kruskal-Wallis one-way ANOVA followed by post hoc Dunn’s multiple comparisons (**f**)). **g**, Representative β-catenin confocal immunofluorescence images in NC cells and VFC T1 and T3 cells subjected to static stretch (three biological replicates). Scale bar 20 µm. **h** & **i**, Quantification of total (**h**) and nuclear (**i**) β-catenin expression (integrated density per cell number in the FoV) for NC and VFC T1 and T3 cells subjected to static stretch (NC, *n* = 18; T1, *n* = 18 and T3, *n* = 18; pooled from three biological replicates; Two-tailed paired t-test). **j**, Relative RET mRNA expression (RNA-seq data; gene count normalized to average value across biological replicates) in NC, VFC T1 and T3 cells (three biological replicates). Data are mean ± s.d,. Box plots are Tukey box plots and show the median and interquartile range (IQR) and whiskers extend to 1.5 times the IQR. Related to Fig. 4. Source data

Prompted by the striking cell-intrinsic differences in adhesive and mechanical properties between VFC and NC cells, we sought to determine if these alterations extended to the cellular response to mechanical stimuli. To recapitulate the mechanical forces in the vocal folds, we subjected the cells to two types of mechanical stimulus: stretching to mimic opening and closing of the vocal folds, and vibration, which occurs during phonation. The uniaxial cyclic stretching of cells (1 Hz, 20% stretch) for 1 h induced alignment (coherency) of the NC and T1 cancer cells perpendicular to the stretch direction, as exemplified by the visualization of actin filaments and phosphorylated myosin light chain (pMLC; Fig. 4a,b and Extended Data Fig. 5a,b). The poorly organized T3 cell monolayers did not show visible alignment, even though their actin alignment (coherency) was significantly increased similarly as in NC and T1 cells (Fig. 4b). For the vibration, we chose a stimulus matching the frequency of the human adult vocal fold during normal phonation (50–250 Hz, 1 min off/on)^62^. This induced actin stress fibres (Fig. 4c) and caused a marked remodelling of the monolayer. Furthermore, continued vibration for 6 h induced a significant increase in the extrusion of highly contractile, pMLC-positive cells in the T3 VFC, but not in the NC or T1 cells (Fig. 4d,e and Extended Data Fig. 5c). This suggests that vocal-fold-like mobility in the T3 cell layer induces cell extrusion akin to cell ejection from crowded epithelia as a mechanism to ensure epithelial homeostasis and epithelium integrity^63^.

## Mechanical stimuli downregulate oncogenic nuclear β-catenin

**Extended Data Fig. 6:**
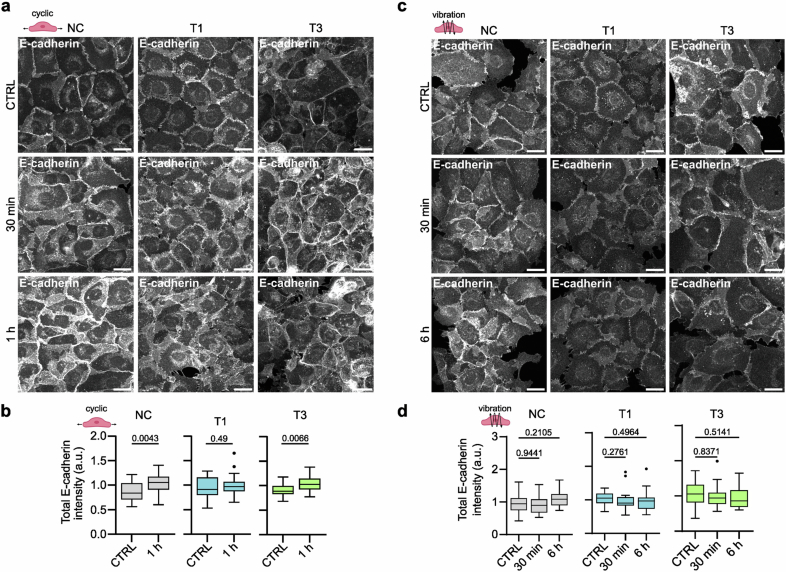
Mechanical stretching increases total E-cadherin levels. **a**, Representative E-cadherin confocal immunofluorescence images of NC and VFC T1 and T3 cells subjected to cyclic stretch for 1 h compared to non-stretched control (three biological replicates). Scale bar 25 µm. **b**, Quantification of total E-cadherin expression (integrated density per number of nuclei in field of view (FoV)) in NC and VFC T1 and T3 cells subjected to cyclic stretch for 1 h compared to non-stretched control (NC *n* = 18, T1 *n* = 18 and T3 *n* = 18; pooled from three biological replicates). Ordinary one-way ANOVA followed by Dunnett’s multiple comparisons test was used to assess statistical significance for NC and T3 cells. Kruskal-Wallis test followed by Dunn’s multiple comparisons test was used for T1 cells. Scale bar 25 µm**. c**, Representative E-cadherin confocal immunofluorescence images of NC cells and VFC T1 and T3 cells subjected to vibration (50-250 Hz, 1 min on/off) for 30 min or 6 h compared to non-vibrated control (three biological replicates). Scale bar 25 µm. **d**, Quantification of E-cadherin expression (integrated density per number of nuclei in FoV) in NC and VFC T1 and T3 cells subjected to vibration for 30 min or 6 h compared to non-vibrated control (NC, *n* = 18; T1, *n* = 18 and T3, *n* = 18; pooled from three biological replicates). Ordinary one-way ANOVA followed by Dunnett’s multiple comparisons test was used to assess statistical significance. Box plots are Tukey box plots and show the median and interquartile range (IQR) and whiskers extend to 1.5 times the IQR. Related to Fig. 4. Source data

Next, we investigated whether mechanical manipulation would cause changes in cell–cell junctions. Before stimulation, we noticed that β-catenin was significantly more nuclear in T1 and T3 cells compared with the NC cells (Fig. 4f,g,i,j). This was particularly interesting, since nuclear β-catenin acts as a transcription factor activating signalling pathways that promote tumour formation^64,65^. Uniaxial cyclic stretching (1 Hz, 20% stretch) for 1 h caused the alignment of β-catenin-positive junctions in NC and T1 cells (Extended Data Fig. 5d), and a significant reduction in nuclear and total β-catenin levels in T1 and T3 cells (Fig. 4f,h and Extended Data Fig. 5e), which was also evident in the vibrated cells (Fig. 4i,k and Extended Data Fig. 5f). Similar to cyclic stretch, 20% static stretch for 30 min and 1 h significantly reduced nuclear β-catenin levels in the T1 and T3 cells. However, the total β-catenin was reduced significantly only in T3 cells (Extended Data Fig. 5g–i). In colorectal carcinoma, mechanical stimuli have been shown to regulate β-catenin via RET kinase^27^; however, RET expression was very low and remained unchanged between NC and VFC T1 and T3 cells, suggesting it is unlikely to be a key regulator of β-catenin in this cancer type (Extended Data Fig. 5j). These mechanical-manipulation-induced changes were specific to β-catenin, as the E-cadherin levels were not altered by vibration and were modestly upregulated by cyclic stretch in NC and T3 cells (Extended Data Fig. 6a–d). Collectively, these data indicate that the cellular mechanoresponses under stretch or vibration are different between NC and VFC cells, as well as the mechanical stimulation of T3 cells, which represent the mechanically immobile stage of VFC in vivo, and triggers cell extrusion and downregulation of oncogenic nuclear β-catenin.

## Mechanical stimuli decrease nuclear and total YAP levels

**Fig. 5:**
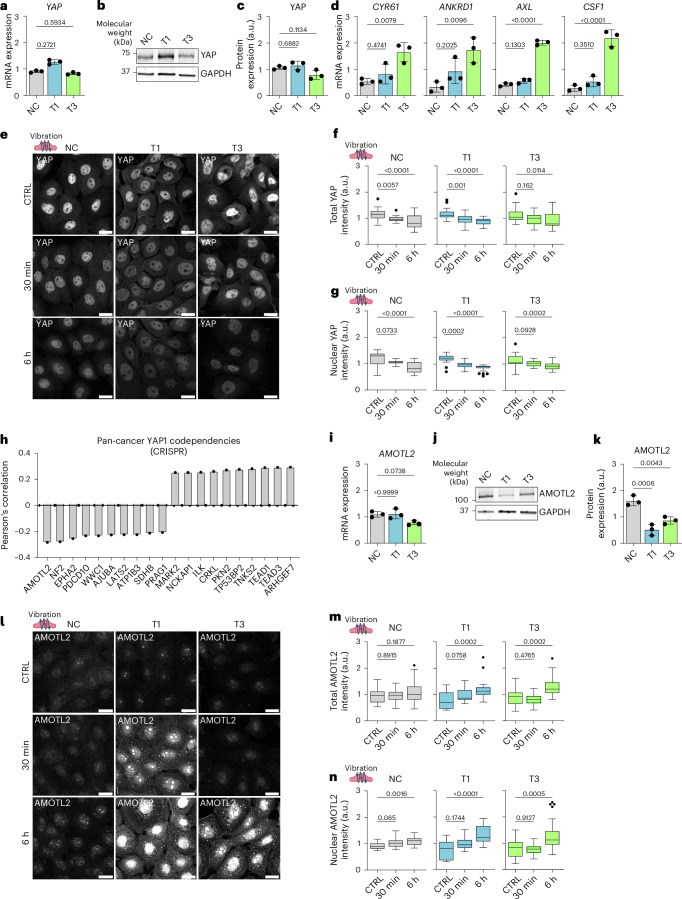
Mechanical stimuli decrease nuclear and total YAP levels. **a**, Relative *YAP* mRNA expression (gene count) in NC and VFC T1 and T3 cells (mean ± s.d.; three biological replicates). **b**,**c**, Representative immunoblot (**b**) and quantification (**c**) of YAP protein expression in NC and VFC T1 and T3 cells (mean ± s.d.; three biological replicates). **d**, Relative YAP target gene mRNA expression in NC and VFC T1 and T3 cells (mean ± s.d.; three biological replicates). **e**, Representative YAP confocal images in NC and VFC T1 and T3 cells ± vibration (50–250 Hz, 1 min on/off; three biological replicates). **f**,**g**, Quantification of total (**f**) and nuclear (**g**) YAP expression (integrated density per number of nuclei in FoV) in NC and VFC T1 and T3 cells ± vibration (*n* = 24 FoVs for each condition pooled from three biological replicates). **h**, Quantification of pan-cancer *YAP1* CRISPR codependency (DepMap) as Pearson’s correlation. Data represent the mean. **i**, Relative *AMOTL2* mRNA expression in NC and VFC T1 and T3 cells (mean ± s.d.; three biological replicates). **j**,**k**, Representative immunoblot (**j**) and quantification (**k**) of relative AMOTL2 protein expression in NC and VFC T1 and T3 cells (mean ± s.d.; three biological replicates). **l**, Representative AMOTL2 confocal images in NC and VFC T1 and T3 cells ± vibration (three biological replicates). **m**,**n**, Quantification of total (**m**) and nuclear (**n**) AMOTL2 expressions (analysed as in **g**) in NC and VFC T1 and T3 cells ± vibration (*n* = 24 FoVs for each condition pooled from three biological replicates). Tukey box plots show the median and IQR. Whiskers extend to 1.5 times the IQR. One-way Kruskal–Wallis test followed by Dunn’s multiple comparisons (**a**, **f**–**h**, **m** and **n**) and ordinary one-way ANOVA followed by Dunnett’s multiple comparisons (**c**, **d** and **k**). Scale bars, 20 μm. Source data

**Extended Data Fig. 7:**
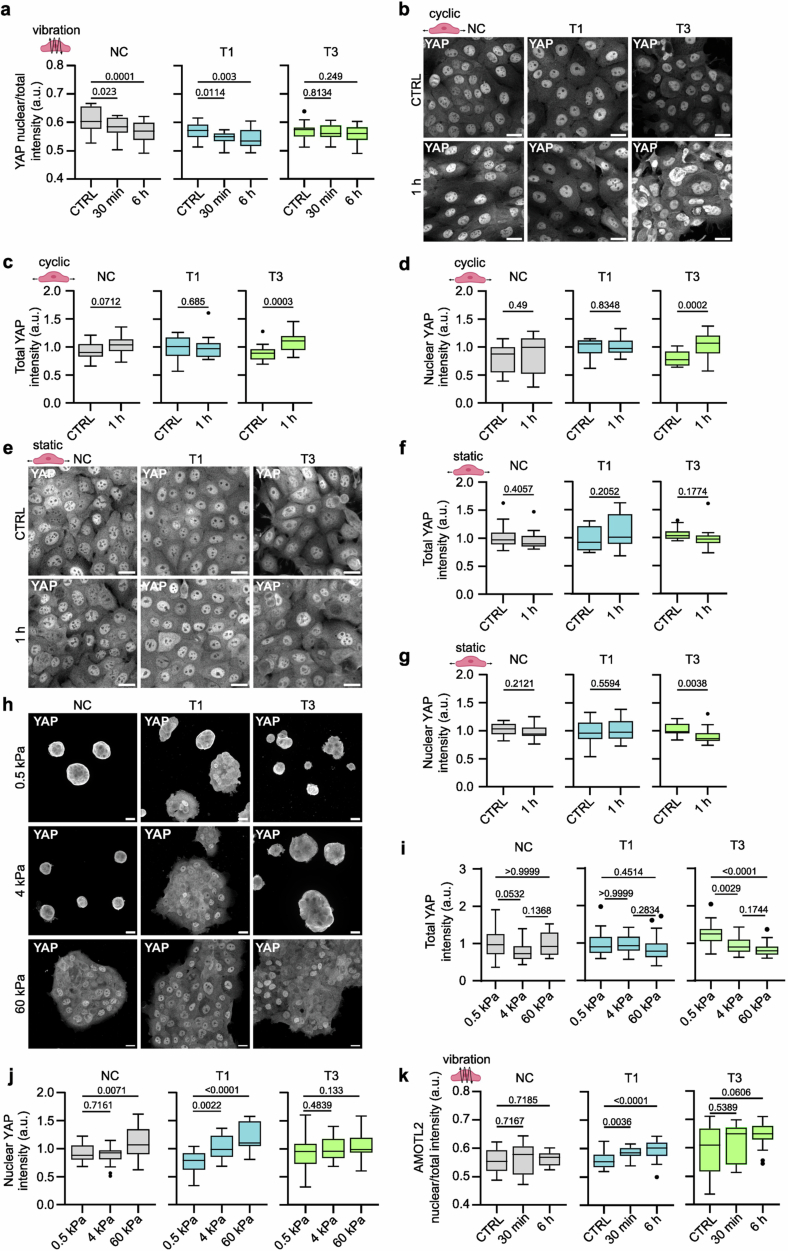
Mechanical stimuli decrease nuclear and total YAP levels. **a**, Quantification of nuclear YAP expression relative to total YAP (average integrated density per cell in the field of view (FoV)) in NC and VFC T1 and T3 cells subjected to vibration (50-250 Hz, 1 min on/off) for 30 min or 6 h compared to non-vibrated control (NC *n* = 24, T1 *n* = 24, T3 *n* = 24; pooled from three biological replicates). Ordinary one-way ANOVA followed by Dunnett’s multiple comparisons test was used to assess statistical significance. **b**, Representative YAP confocal immunofluorescence images of NC cells and VFC T1 and T3 cells subjected to cyclic stretch for 1 h compared to non-vibrated control (three biological replicates). Scale bar 25 µm. **c** & **d**, Quantification of total (**c**) and nuclear (**d**) YAP expression (integrated density per number of nuclei in FoV) in NC and VFC T1 and T3 cells subjected to cyclic stretch for 1 h compared to non-vibrated control (NC *n* = 18, T1 *n* = 18 and T3 *n* = 18; pooled from three biological replicates). Two-tailed unpaired t-test was used to assess statistical significance. **e**, Representative YAP confocal immunofluorescence images of NC and VFC T1 and T3 cells subjected to static stretch for 1 h compared to non-vibrated control (three biological replicates). Scale bar 25 µm. **f** & **g**, Quantification of total (**f**) and nuclear (**g**) YAP expression (integrated density per number of nuclei in FoV) in NC and VFC T1 and T3 cells subjected to static stretch for 1 h compared to non-vibrated control (NC *n* = 18, T1 *n* = 18 and T3 *n* = 18; pooled from three biological replicates). Two-tailed unpaired *t*-test was used to assess statistical significance. **h**, Representative YAP confocal immunofluorescence images of NC and VFC T1 and T3 cells on 0.5 kPa, 4 kPa and 60 kPa hydrogels (three biological replicates). Scale bar 25 µm. **i** & **j**, Quantification of total (**i**) and nuclear (**j**) YAP expression (integrated density per number of nuclei in FoV) in NC and VFC T1 and T3 cells on 0.5 kPa, 4 kPa and 60 kPa hydrogels (NC, *n* = 25 (0.5 kPa), 24 (4 kPa) and 25 (60 kPa); T1, *n* = 24 (0.5 kPa), 26 (4 kPa) and 24 (60 kPa); *n* = 25 (0.5 kPa), 25 (4 kPa) and 25 (60 kPa); pooled from three biological replicates). One-way Kruskal-Wallis test followed by Dunn’s multiple comparisons test (i) and ordinary one-way ANOVA followed by Dunnett’s multiple comparisons test (j) used to assess statistical significance. **k**, Quantification of AMOTL2 nuclear expression relative to total AMOLT2 in NC cells and VFC T1 and T3 cells subjected to vibration (50-250 Hz, 1 min on/off) for 30 min or 6 h compared to non-vibrated control (NC *n* = 24), T1 *n* = 24 and T3 *n* = 24; pooled from three biological replicates). Ordinary one-way ANOVA followed by Dunnett’s multiple comparisons test was used to assess statistical significance in NC and T1 cells. Kruskal-Wallis test followed by Dunn’s multiple comparisons test was used to assess statistical significance in T3 cells. Box plots are Tukey box plots and show the median and IQR and whiskers extend to 1.5 times the IQR. Related to Fig. 5. Source data

In addition to β-catenin, another key mechanosensitive oncoprotein in cancer is YAP, which shuttles between the cytoplasm and nucleus, where it can activate downstream signalling pathways that maintain oncogenic signalling cascades^66^. Total YAP RNA (Fig. 5a) and protein (Fig. 5b,c) expression levels showed no significant differences in VFC cell lines, compared with NC cells, but the RNA expression of YAP downstream targets cysteine-rich angiogenic inducer 61 (*CYR61*), Ankyrin Repeat Domain 1 (*ANKRD1*), *AXL* and macrophage colony-stimulating factor (*CSF1*) were increased in T3 VFC cells (Fig. 5d), suggesting elevated pathway activity. Importantly, vibration decreased the total and nuclear YAP levels in a time-dependent manner with prolonged vibration (6 h), similar to the changes observed for β-catenin, having a more significant effect than the acute 30-min stimulation (Fig. 5e–g). Concordant with these kinetics, the nuclear to total ratio, which is under acute mechanical control in many cell types, was less prominent and not significant in T3 cells (Extended Data Fig. 7a). Interestingly, uniaxial cyclic stretching (1 Hz, 20% stretch) for 1 h significantly increased the YAP levels in T3 cells and had no effect in NC and T1 cells (Extended Data Fig. 7b–d). On the other hand, static stretch (20% for 30 min or 1 h) significantly reduced the YAP levels in T3 cells, again having no effect on NC and T1 cells (Extended Data Fig. 7e–g). To investigate this further, we plated cells on hydrogels of different rigidities (0.5, 4 or 60 kPa) to simulate a form of permanent strain on the stiffer substrate. NC and T1 cells responded as expected, with the nuclear YAP (but not total YAP) intensity rising with increasing stiffness. By contrast, T3 cells displayed a reduction in the total YAP levels but no significant change in nuclear YAP with increasing rigidity (Extended Data Fig. 7h–j). These data imply that β-catenin and YAP have distinct responses to cell stretching but are similarly downregulated by cell vibration. Furthermore, vibration appears to primarily regulate YAP levels, rather that YAP mechanoresponsive shuttling to the nucleus.

## Mechanical stimuli increase AMOTL2 protein levels

To further understand the role of YAP in squamous cell carcinoma, we surveyed YAP1 cancer dependency maps on DepMap^67^. A pan-cancer search identifying the top-20 codependencies in the CRISPR DepMap Public 23Q2+Score Chronos dataset found the strongest dependency hits (Pearson’s correlation, *r*) with Rho Guanine Nucleotide Exchange Factor 7 (ARHGEF7; *r* = 0.29), TEA Domain Transcription Factor 3 (TEAD3, *r* = 0.29), TEA Domain Transcription Factor 1 (TEAD1, *r* = 0.29), Tankyrase 2 (TNKS2, *r* = 0.28) and Angiomotin-like protein 2 (AMOTL2, *r* = –0.28; Fig. 5h). Moreover, ILK, which had increased FA localization in cancer cells, was one of the top-10 positive dependency hits (ILK, *r* = 0.26; Extended Data Fig. 2g,h).

Intrigued by these findings, we sought to investigate the relationship between YAP and AMOTL2 in our cell model. AMOTL2 is a negative YAP regulator and has been shown to directly interact with YAP, retaining it within the cytoplasm^68–71^. *AMOTL2* RNA levels were not significantly different between the cell lines (Fig. 5i). However, AMOTL2 protein levels were significantly lower in VFC cells compared with NC cells (Fig. 5j,k). Vibration significantly increased AMOTL2 total and nuclear levels in VFC cells (Fig. 5l–n and Extended Data Fig. 7k), coinciding with a decrease in YAP levels (Fig. 5f,g). In summary, these results suggest that mechanical stimulation may decrease oncogenic nuclear YAP levels through an AMOTL2-dependent regulatory mechanism and the findings further support the notion of tissue mechanics contributing to vocal fold homeostasis, playing an anti-oncogenic role in VFC.

## High ECM score correlates with poor patient survival and YAP

**Fig. 6:**
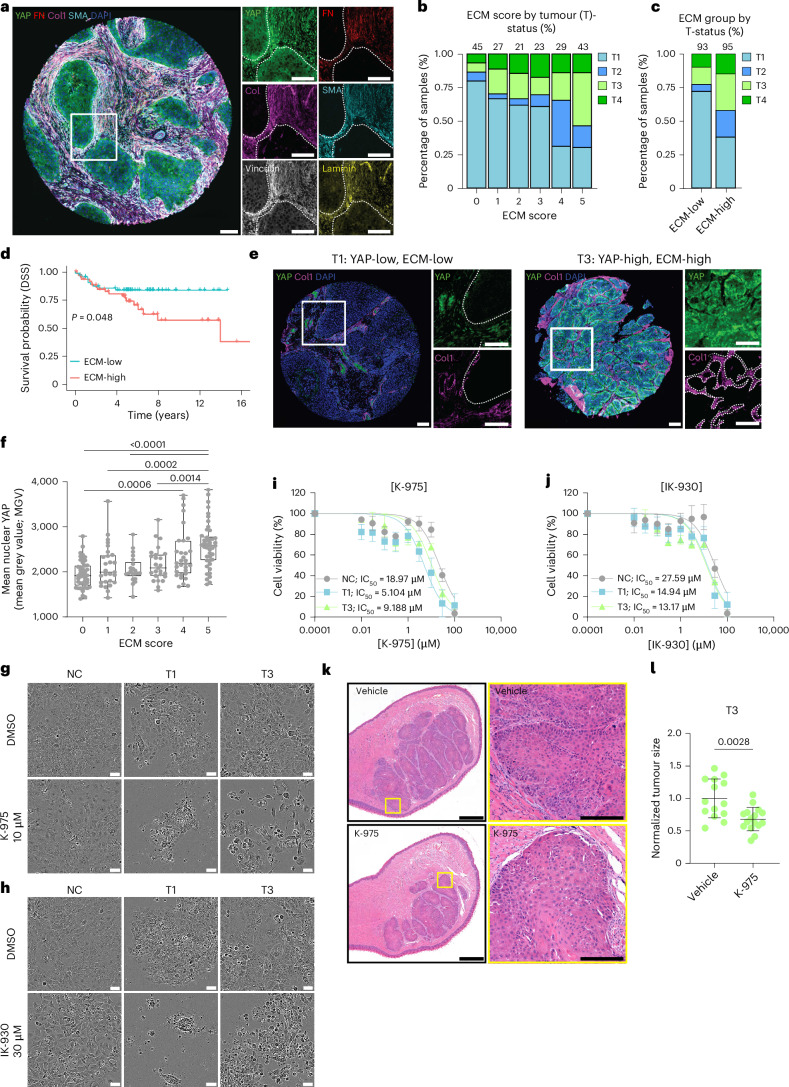
High YAP correlates with high ECM expression and poor disease-specific survival. **a**–**f**, Analysis of patient TMA samples (*n* = 193, unless indicated otherwise). **a**, Representative composite immunofluorescence images of a TMA core stained as indicated. **b**, Quantification of the relationship between stromal ECM score (median patient-level expression of stromal fibronectin (FN), collagen I (ColI), SMA, vinculin and laminin) and tumour size (T-status) in TMA-multiplexed immunohistochemistry illustrated as a percentage of the samples (number of tumour samples indicated above; five samples excluded due to no available tumour staging). **c**, ECM group (ECM-low and ECM-high) by T-status illustrated as a percentage of the samples (number of tumour samples indicated above). **d**, Disease-specific survival of ECM-high and ECM-low patients. Log-rank test for Kaplan–Meier analysis. **e**, Representative YAP and ColI staining in a YAP-low/ECM-low T1 tumour and a YAP-high/ECM-high T3 tumour. **f**, Correlation analysis between the stromal ECM score and mean nuclear YAP expression in the tumour epithelium. The box plot shows the median and IQR. Whiskers extend to the minimum and maximum values. One-way ANOVA with Tukey’s multiple comparisons (**a** and **d**: white boxes denote regions of higher magnification displayed as single-channel insets; dotted lines indicate the tumour–stroma border). Scale bars, 100 μm. **g**–**j**, Representative phase contrast images (**g** and **h**) and viability (**i** and **j**) of NC and VFC T1 and T3 cells treated with YAP-TEAD inhibitors, K-975 (**g** and **i**) or IK-930 (**h** and **j**) for 48 h (four biological replicates; mean ± s.e.m. with a nonlinear fit; log_10_ scale). Scale bar, 50 µm. **k**,**l**, Representative haematoxylin and eosin staining (**k**) and quantification of normalized tumour size (**l**) in vehicle and K-975-treated mice bearing a tongue tumour (VFC T3 cells; mean ± s.d.; *n* = 14 (vehicle) and n = 16 (K-975) samples pooled from four mice per condition; two-tailed Mann–Whitney test). Scale bar, 1 mm; 200 μm (zoomed-in view). Source data

**Extended Data Fig. 8:**
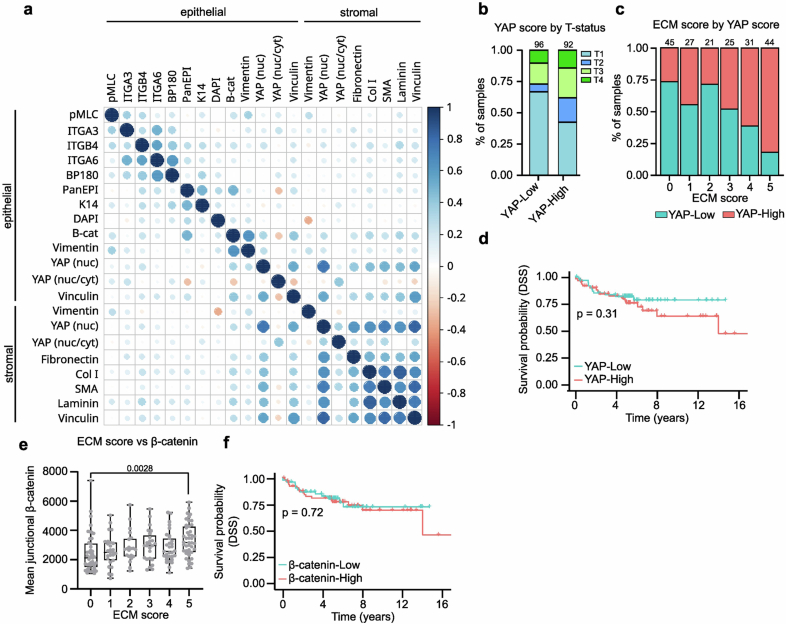
High YAP levels correlate with high ECM expression and poor disease specific survival. **a-f**, Analysis of patient TMA samples. Unless otherwise indicated, total *n* = 193 patient samples. **a**, Patient-level correlation of epithelial (pMLC, ITGA3, ITGB4, ITGA6, BP180, PanEpi, K14, DAPI, vimentin, YAP and vinculin) and stromal (vimentin, YAP, fibronectin, ColI, SMA, laminin and vinculin) marker mean expression in multiplex-stained TMAs. **b**, Epithelial YAP score by T-status illustrated as percentage of samples (%) (no. of samples indicated above each bar; five patient samples were excluded due their tumour stage being defined as NA). **c**, Stromal ECM score by epithelial YAP score illustrated as percentage of samples (%). **d**, Disease specific survival (DSS) of YAP-high (*n* = 97) and YAP-low (*n* = 96) patients. Kaplan-Meier analysis with Log-rank test was used to assess statistical significance. **e**, Quantification of the relationship between stromal ECM-score and mean junctional β-catenin expression in the tumour epithelium. Box plot shows median and IQR. Whiskers extend to min and max values. One-way ANOVA with Tukey’s multiple comparisons test. All p-values from comparisons between conditions are shown in the source data. Only the significant p-value is shown here. **f**, DSS of β-catenin-high (*n* = 99) and β-catenin-low patients (*n* = 94). Kaplan-Meier analysis with Log-rank test was used to assess statistical significance. Related to Fig. 6. Source data

To translate the in vitro findings into a more clinically relevant setting, we investigated the in vivo relevance of the identified mechanoregulators using multiplexed immunohistochemistry and patient cohorts. We generated a custom laryngeal cancer tissue microarray (TMA) with cancer patient samples from T1 to T4 (*n* = 193). We first noticed that there is a high correlation between all stromal ECM proteins (Extended Data Fig. 8a) and, therefore, implemented an ECM score, which considers median intensity values for all the ECM and ECM-related proteins (fibronectin, collagen I, SMA, laminin and vinculin) in the tumour stroma across the patient cohort. Each patient was assigned an ECM score based on how many of the five ECM proteins were expressed at above-average levels, with scores ranging from 0 (all ECM and ECM-related proteins below average) to 5 (all ECM and ECM-related proteins above average). Scores of 0–2 were then classed as ‘ECM-low’, whereas scores of 3–5 were classed as ‘ECM-high’. The analysis revealed a notable correlation between the ECM score and T-status, with lower ECM scores being associated with lower T-status (Fig. 6a–c), and a significant correlation with patient survival (Fig. 6d).

**Extended Data Fig. 9:**
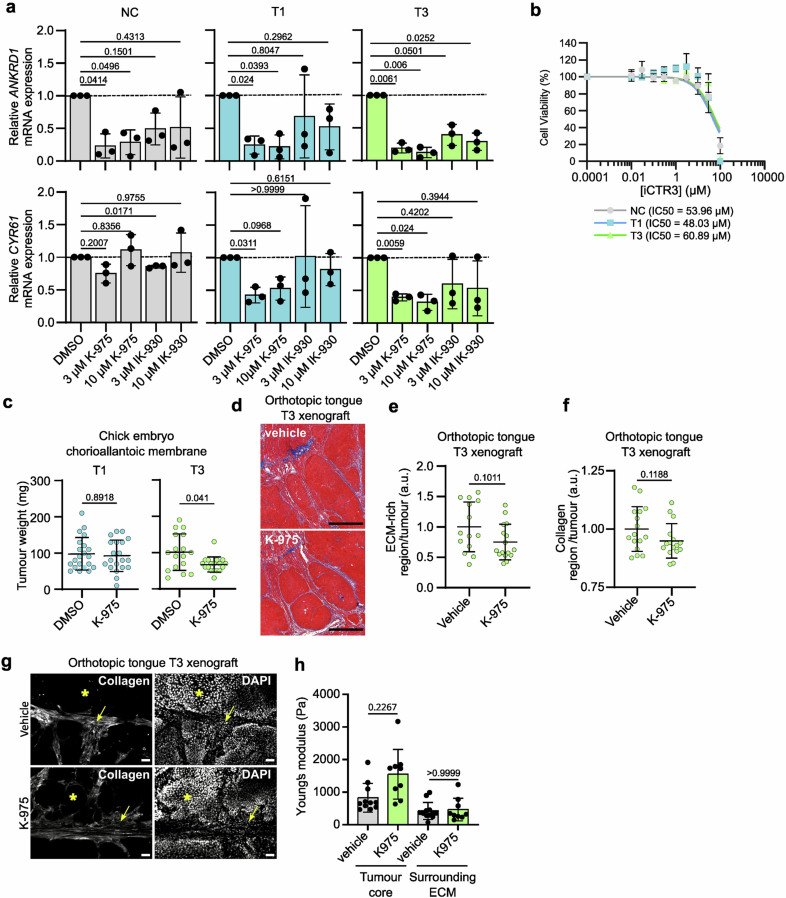
YAP-TEAD inhibition in in vitro and in vivo models. **a**, Relative *ANKRD1* and *CYR61* mRNA expression in NC cells and VFC T1 and T3 cells after inhibition of YAP-TEAD pathway with K-975 or IK-930 inhibitors for 48 h compared to DMSO control (three biological replicates). Data are mean ± s.d. One-way ANOVA followed by Dunnett’s multiple comparisons test was used to assess statistical significance. **b**, Viability of NC and VFC T1 and T3 cells treated with wnt/β-catenin inhibitor iCRT3 (three biological replicates). Data are mean ± standard error of the mean with a non-linear fit; log_10_ scale). **c**, Quantification of tumour weight (mg) in DMSO and K-975 (10 µM) inhibitor-treated VFC T1 and T3 cells in a CAM model (T1 *n* = 21 (DMSO) and 20 (K975); T3 *n* = 17 (DMSO) and 18 (K975). Data are mean ± s.d.). Two-tailed Mann-Whitney test was used to assess statistical significance. **d**, Representative Masson’s trichrome stained tumours in vehicle or K-975-treated mice bearing a tongue tumour (VFC T3 cells) (*n* = 4 mice per condition). Scale bar 500 µm. **e**, Quantification of ECM-rich regions per tumour in vehicle or K-975-treated mice bearing a tongue tumour (VFC T3 cells) (*n* = 4 mice per condition). Data are dot plots ± s.d. Two-tailed Mann-Whitney test was used to assess statistical significance. **f**, Quantification of collagen area (Masson’s trichrome staining) per tumour in vehicle (*n* = 4) and K-975 (*n* = 4) treated mice bearing a tongue tumour (VFC T3 cells). Data are mean ± s.d. Two-tailed Mann-Whitney test was used to assess statistical significance. **g**, Representative collagen (CNA-35) and DAPI stained tumours (T3 cells) in vehicle or K-975-treated mice bearing a tongue tumour (VFC T3 cells) (*n* = 4 mice per condition). Scale bar 100 µm. **h**, Quantification of tumour stiffness (Young’s modulus, Pa) in vehicle or K-975-treated mice bearing a tongue tumour (VFC T3 cells) (*n* = 4 mice per condition. One cryosection per mouse was used to realize 2-3 measurements in different tumour regions. Each dot represents one measurement). Data are mean ± s.d. One-way Kruskal-Wallis test was used to assess statistical significance. Related to Fig. 6. Source data

To determine whether YAP expression correlates with T-status (Extended Data Fig. 8b) and ECM score (Extended Data Fig. 8c), we calculated the median per-patient nuclear YAP value within the tumour epithelium, and classified samples as either YAP-high or YAP-low based on this threshold. We found that patient-level nuclear YAP levels (intensity) increased significantly with higher ECM scores in tumours (Fig. 6e,f). YAP-high tumours tended to have lower patient survival and higher staging (Extended Data Fig. 8b–d). Junctional β-catenin levels were slightly higher in samples with the highest ECM score compared with those with the lowest ECM score, but β-catenin levels in patient samples were not predictive of patient survival outcomes (Extended Data Fig. 8e,f). Having established that patients with high ECM scores in the stroma have higher nuclear YAP in their tumour and a worse clinical outcome, we set out to explore whether the inhibition of YAP-TEAD would affect cell viability. Treating cells with a YAP-TAZ-TEAD inhibitor, K-975, which covalently binds to a palmitate-binding pocket of TEAD and inhibits YAP function^72^, resulted in a significant downregulation of YAP downstream targets *CYR61* and *ANKRD1* mRNA levels, indicative of an on-target effect (Extended Data Fig. 9a) and a dose-dependent decrease in cell viability, with the T3 VFC cells showing the highest sensitivity to the drug. Another YAP-TAZ-TEAD inhibitor, IK-930, which is in phase I clinical trials for advanced solid tumours^73^, also showed increased sensitivity in VFC cells (Fig. 6g–j). By contrast, the NC and VFC cells were relatively insensitive to Wnt and β-catenin-responsive transcription inhibitor iCTR3 (refs. ^74,75^ and Extended Data Fig. 9b).

Importantly, this K-975 sensitivity was also observed in vivo in T3 but not T1 cancer xenografts models in the chick embryo chorioallantoic membrane (CAM) assay in fertilized eggs (Extended Data Fig. 9c). Furthermore, the YAP-TAZ-TEAD inhibitor treatment of mice with T3 tumours in the tongue (orthotopic transplantation) significantly limited tumour growth compared with control treatment (Fig. 6k,l), without significantly impacting collagen deposition or tumour rigidity (Extended Data Fig. 9d–h). Taken together, these findings reveal the clinical potential for YAP-TAZ-TEAD inhibition as a treatment option for VFC.

## Outlook

Cells sense the biophysical features of their surrounding tissue, and the ensuing biomechanical signalling controls epithelial homeostasis, malignant progression, directed cell migration and drug sensitivity^12,76–78^. The vast majority of research in this area, however, draws from solid carcinomas arising from immobile tissue, such as the mammary gland, and the role of altered tissue mechanics in homeostasis and oncogenic properties of constantly moving epithelia remain poorly understood. Here we used cell culture models to recapitulate the key features of vocal fold epithelia, including ECM rigidity, tissue stretching and vibration. We show that concordant with the vocal fold epithelia becoming mechanically fixed and invasive with increasing T-status, VFC upregulates the expression of multiple ECM components, is stiffer than normal vocal fold and proliferates in a stiffness-dependent manner. Unlike kinetically arrested, densely packed (jammed) NC squamous epithelia, patient-derived VFC cells are in a flocking, hyper-motile state, similar to the one previously established for invasive breast carcinomas^49,51^, in line with their high invasive capacity. We acknowledge that our two-dimensional model system to mechanically stimulate cells does not fully recapitulate the in vivo situation. Although the healthy vocal fold epithelium in vivo is primarily a two-dimensional monolayer adhering to the basement membrane, tumour progression disrupts this and the cells transition towards a more 3D environment.

Cell cycle re-entry of arrested epithelia is regulated by nuclear translocation and transcriptional activity of YAP and β-catenin^79^. Malignant head and neck squamous cell carcinoma tissues have higher YAP1 expression compared with benign patient samples, and YAP1 activation drives oral squamous cell carcinoma tumourigenesis and correlates with poor patient survival^80–83^. However, YAP and β-catenin have not been explored in the molecularly distinct VFC (ref. ^84^). We find that mechanical vibration, mimicking normal-like vocal fold mobility, downregulates nuclear YAP levels with a concomitant induction of the YAP inhibitor AMOTL2 in VFC cells derived from increasingly immobile tumours^85^. Moreover, high YAP correlates with higher tumour staging and higher stromal ECM content, which, in turn, correlates with poor clinical outcome in patient samples. VFC cells are increasingly sensitive to clinically tested^73^ YAP-TEAD small-molecule inhibitors both in vitro and in vivo. Thus, normal tissue mechanics, mimicked in our cell culture systems by stretching and vibration, downregulate the activity of two relevant and synergistically acting oncogenic pathways^79^. Although our clinical evaluation comprises hundreds of patient samples, it is important to acknowledge that the small number of cell lines used in the functional studies limits the generalizability of the findings and may not fully capture the heterogeneity of VFC.

These insights into the role of tissue mobility in maintaining homeostasis and suppression of malignancy may extend to other carcinomas arising from mobile epithelia and broaden our horizon on the mechanical control of cancer progression.

## Methods

### Ethical considerations

Use of animals: we confirm that our research complies with all relevant ethical regulations. Mouse intratongue injection was ethically assessed and authorized by the National Animal Experiment Board and in accordance with The Finnish Act on Animal Experimentation (animal license number ESAVI/6253/2024). All efforts were made to minimize animal suffering and to reduce the number of animals used. All experiments respected the maximum tumour diameter (15 mm) permitted by the authorization bodies.

Patient data: patient samples were obtained at the Department of Otorhinolaryngology—Head and Neck Surgery at the Turku University Hospital under the Finnish Biobank Act, with written informed consent from the sample donors (§279, 9/2001). On collection, the samples were given an arbitrary identifier and no patient identifiers, excluding age, gender, prior treatments and histopathological features, were available or recorded. Tissue samples were snap frozen with liquid nitrogen and stored at –80 °C until further processing.

The study and utilization of human tissue samples were approved by the Finnish National Authority for Medicolegal Affairs (V/39706/2019), the Institutional Review Board at the Helsinki University Hospital (HUS/745/2021) and a research permission was granted (HUS/85/2021).

Formalin-fixed and paraffin-embedded tumour samples were obtained from the pathology archives of the Helsinki Biobank. Patient consent was waived due to the retrospective nature of the data in accordance with approval from the Finnish National Supervisory Authority for Welfare and Health and the Regional Ethics Committee of the University of Turku. The authors affirm that the study was conducted following the rules of the Declaration of Helsinki of 1975, revised in 2013.

Cell lines: UT-SCC-11 (T1) and UT-SCC-103 (T3) cell lines generated at the Turku University Hospital have undergone scientific evaluation by Auria Biobank with a positive decision of release (AB22-7195) to be used in the study. Use of these cell lines for other purposes requires ethical approval and permission from the Auria Biobank.

### TCGA data acquisition and analysis

TCGA head and neck squamous cell carcinoma dataset was retrieved and filtered for patient IDs with laryngeal cancer as the primary tumour site. Pathology reports were then reviewed to assess the tumour subsite and the involvement of vocal folds. Raw files were downloaded from the Xena browser (https://xenabrowser.net/). Differentially expressed genes (log_2_[fold change]) were assessed using Bioconductor R package reproducibility-optimized test statistic (ROTS; v1.14.0), defining genes with FDR < 0.05 as differentially expressed^87^. GO was performed using clusterProfiler (v4.8.3) in R^88^.

### TMA

TMA blocks with duplicate core biopsies were prepared from the formalin-fixed and paraffin-embedded samples using a TMA Grand Master (3DHISTECH) at the Helsinki University Hospital. A total of 198 patients with known TNM staging (a recognised system to describe the extent of the disease, based on tumour characteristics, spreading to nearby lymph nodes and metastasis to other organs) and survival endpoints were included in the study.

### Chick embryo CAM model

The shell of fertilized chicken eggs was cleaned with 70% ethanol before starting development, that is, placing the eggs in a humidified incubator (50% moisture + 37 °C). On day 3 of development, a small hole was made with a needle and tweezers in the eggshell to drop the CAM away from the shell. On developmental day 7, the hole was widened with tweezers to place a plastic ring on the CAM. One million UT-SCC-11 or UT-SCC-103 cells were implanted inside the ring in 20 µl of 50% Matrigel (Corning) diluted in phosphate-buffered saline (PBS) supplemented with control or drug treatment (dimethyl sulfoxide (DMSO) or 10 μM of TEAD inhibitor K-975), after which the hole was covered with a parafilm to avoid drying of the CAM. Tumours were harvested 4–5 days post-implantation by placing the eggs on ice for 30 min before dissecting, weighing and fixing the tumours in 10% phosphate-buffered formalin (pH 7; VWR).

### Intratongue mouse model

Female immunocompromised mice (NOD.Cg-PrkdcscidIl2rgtm1Wjl/SzJ; Charles River) between 9 and 12 weeks of age were treated with a painkiller and anti-inflammatory mixture (0.07 mg kg^−1^ of buprenorphine and 5 mg kg^−1^ of carprofen in 100 ml of PBS injected intraperitoneally) 30 min before tongue injection and twice a day for 3 days after injection. Mice were anaesthetized (75 mg kg^−1^ of ketamine and 6 mg kg^−1^ of xylazine in 200 μl of PBS injected subcutaneously) for injections. A single-cell suspension (30,000 cells) was prepared in 30% growth-factor-reduced Matrigel (Corning) diluted in PBS and was injected into the tongue using syringes (0.3 ml, 0.30 mm (30 G) × 8 mm; BD Micro-Fine). Mice were given softened food ad libitum to ensure that they could eat regardless of tumour growth. On day 7 post-injection, 80 mg kg^−1^ of the K-975 inhibitor (diluted in 10% DMSO (D2650, Sigma), 40% PEG400 (63012, Reidel-de Haen), 5% Tween 80 (P4780, Sigma) and 150 mM of NaCl) was orally administered to mice daily until the animals were killed. Oral gavaging was performed by coating needles in 24% sucrose (diluted in sterile water) to reduce oesophageal irritation^89^. Animal weight and tumour growth were closely monitored for 2 weeks (endpoint). Mice were killed and tongues were collected in 10% phosphate-buffered formalin (FF-Chemicals) or freshly frozen in an optimal cutting temperature compound (Tissue-Tek). Formalin-fixed tissues were embedded in paraffin and 4–5-µm sections were stained with haematoxylin and eosin and Masson’s trichrome using standard protocols. Optimal-cutting-temperature-embedded tissues were used to assess the tissue stiffness using AFM (see the ‘AFM’ section).

### AFM

For all mechanical measurements, tissues were freshly frozen in the optimal cutting temperature compound (OCT) and cut into either 16 or 30 μm cryosections, as indicated, at –20 °C, followed by immediate transfer to poly-L-lysine-coated glass-bottom dishes or slides. Before the measurements, PBS containing complete EDTA-free protease inhibitor (Sigma) was utilized to thaw sections.

Tongue xenograft tumour samples (30 μm cryosections) were incubated with 40 µg ml^−1^ CNA35-GFP to label collagen and 5 µg ml^−1^ DAPI in PBS containing protease inhibitors and 0.2% Triton X-100 for at least 1 hour at room temperature. AFM measurements were done on the same day on a JPK NanoWizard II system (Bruker Nano) with a CellHesion module mounted on a ZEISS LSM510 confocal microscope (Carl ZEISS NTS) utilizing JPK SPM Control Software (v4.2). Patient biopsy (16 μm cryosections) measurements were performed within 30 minutes of tissue thaw on a JPK NanoWizard 4 system (Bruker Nano) with a CellHesion module mounted on an Eclipse Ti2 inverted fluorescence microscope (Nikon) and operated via JPK SPM control software (v6). Silicon nitride cantilevers (nominal spring constant, 0.06 N m^−1^; spherical 4.5-μm-diameter tip; Novascan Technologies) were used to assess xenograft stiffness and MLCT triangular silicon nitride cantilevers (Bruker) were used to measure basement membrane stiffness in patient biopsies. Spring constant and deflection sensitivity were calibrated in fluid via the thermal noise method^90^. All AFM measurements were performed utilizing a 5 × 5 point grid (25 µm × 25 µm). At least five regions were measured per sample. Forces of up to 3 nN were applied at 20 µm s^−1^ constant cantilever velocity. All analyses were performed with JPK Data Processing Software (v4.2 for tongue xenograft tissues and v6 for patient biopsies; Bruker Nano) by first removing the offset from the baseline of raw force curves, then identifying the contact point and subtracting cantilever bending before fitting the Hertz model with the correct tip geometry to determine the Young modulus.

### Cell lines and culture

Spontaneously immortalized human epidermal keratinocyte-derived cell line HaCaT (ref. ^91^), obtained from the collection of BioCity Turku (University of Turku, Finland; referred to as NC cells in this manuscript), UT-SCC-11 (T1 human glottic laryngeal cancer, Turku University Hospital) and UT-SCC-103 (T3 human glottic laryngeal cancer, Turku University Hospital) cells were cultured in Dulbecco’s modified Eagle’s medium (Sigma-Aldrich) supplemented with 10% fetal bovine serum (Sigma-Aldrich), 2 mM of L-glutamine (Sigma-Aldrich) and 1% Minimal Essential Medium non-essential amino acid solution (Sigma-Aldrich) at 37 °C, 5% CO_2_. UT-SCC-11 and UT-SCC-103 cell lines generated at the Turku University Hospital have undergone scientific evaluation by Auria Biobank with a positive decision of release (AB22-7195) to be used in the study. Use of these cell lines for other purposes requires ethical approval and permission from the Auria Biobank. All cell lines were regularly tested for mycoplasma using a MycoAlert Mycoplasma Detection Kit (LT07-418, Lonza) and MycoAlert Assay Control Set (LT07-518, Lonza) to ensure mycoplasma-free culturing. Cells were washed with PBS (Gibco) and detached enzymatically with a 0.25% trypsin-EDTA solution (L0932, Biowest).

### Proliferation assay

Plastic (Corning) or Softwell Easy Coat (Matrigen; stiffness values, 0.5 kPa, 25 kPa and 50 kPa) 24-well plates were coated with 10 μg ml^−1^ of collagen I (C8919, Sigma) and 10 μg ml^−1^ of fibronectin (341631, Sigma) diluted in PBS or 10 μg ml^−1^ of growth-factor-reduced Matrigel (354230, Corning) diluted in PBS, at 37 °C for 1 h. Coated plates were washed three times with PBS before seeding 10,000 cells in a culture medium (approximately 5,000 cells cm^−^^2^). Time-lapse live imaging was performed using Incucyte S3 or ZOOM Live-Cell Analysis System for 96 h with 2-h imaging intervals (×10 objective). The medium was changed every second day.

### Migration assay

Softwell Easy Coat (Matrigen) 50-kPa 24-well plates were coated with 10 μg ml^−1^ of collagen I (C8919, Sigma) and 10 μg ml^−1^ of fibronectin (341631, Sigma) diluted in PBS, at 37 °C for 1 h. The coated plates were washed three times with PBS before seeding 1,000 cells in the culture medium (approximately 500 cells cm^−^^2^). Time-lapse live imaging was performed using Nikon Eclipse Ti2-E (×10/0.3 objective) for 24 h with 10-min imaging intervals. Single-cell tracking was performed using TrackMate plugin in ImageJ (National Institutes of Health).

### Invasion assay

Here 200,000 cells were seeded in a serum-free medium on Matrigel transwell inserts (354480, Corning) and placed in the culture medium (approximately 666,000 cells cm^−^^2^). After 45 h of invasion, uninvaded cells in the inner well were removed with cotton buds and invaded cells were fixed with 4% paraformaldehyde diluted in PBS for 10 min at room temperature (RT). Inserts were washed three times with PBS and stained overnight with 4′,6-diamidino-2-phenylindole (DAPI). Invaded cells were assessed by confocal imaging (3i Marianas CSU-W1; ×20/×0.8 objective), quantifying the number of invaded cells per FoV in ImageJ (National Institutes of Health).

### Viability assay

Here 5,000 cells were seeded in a 96-well plate in the culture medium (approximately 15,000 cells cm^−^^2^). DMSO (D265, Sigma) or YAP-TAZ-TEAD inhibitors K-975 (HY-138565, MedChemExpress) or IK-930 (HY-153585, MedChemExpress) and Wnt/β-catenin inhibitor iCRT3 (HY-103705, MedChemEpress) were added at concentrations of 10 nM, 30 nM, 100 nM, 300 nM, 1 µM, 3 µM, 10 µM, 30 µM and 100 µM the following day. Relative cell viability was measured as absorbance at 450 nm after 2-h incubation with a cell counting kit at 37 °C as per the manufacturer’s instructions (Cell Counting Kit-8, ab228554) 48 h after the addition of inhibitor treatment.

### Western blotting

Cells were kept on ice and washed with cold PBS and lysed with heated (90 °C) TX-lysis buffer (50 mM of Tris-HCl, pH 7.5, 150 mM of NaCl, 0.5% Triton X, 0.5% glycerol, 1% SDS, complete protease inhibitor (Sigma-Aldrich), and PhosSTOP tablet (Sigma-Aldrich)). Lysed cells were scraped into an Eppendorf tube and boiled for 5 min at 90 °C followed by 10 min of sonication and 10 min of centrifugation at 16,200*g* at 4 °C in a microcentrifuge. Protein concentrations were determined from the supernatant with DC Protein assay (Bio-Rad) as per the manufacturer’s instructions. Samples were boiled at 90 °C for 5 min before protein separation using precast sodium dodecyl sulfate–polyacrylamide gel electrophoresis gradient gels (4%–20% Mini-PROTEAN TGX, Bio-Rad) and transferred onto nitrocellulose membranes with the semi-dry Trans-Blot Turbo Transfer System (Bio-Rad). Membranes were blocked with AdvanBlock-Fluor blocking solution (AH Diagnostics) diluted 1:1 in PBS for 1 h at RT and incubated overnight at 4 °C, with the primary antibodies diluted in an AdvanBlock-Fluor blocking solution. Membranes were washed for 5 min three times with Tris-buffered saline and 0.1% Tween 20, and incubated 1:2,500 with fluorophore-conjugated Azure secondary antibodies (AH Diagnostics) in the blocking solution for 1 h at RT. Membranes were washed three times with Tris-buffered saline and 0.1% Tween 20 for 5 min at RT. Membranes were scanned using an infrared imaging system (Azure Sapphire RGBNIR Biomolecular Imager), and the band intensities were analysed using Image Studio Lite (Licor) by normalizing the signal to GAPDH or HSP70, which were used as loading controls.

The list of antibodies is provided in Supplementary Table 1.

### PIV analysis

A custom PIV algorithm was developed in Python to measure the cell velocities within monolayers and derive different indicators of cellular motility. Velocity fields were first extracted by processing sequences of images. In short, each image is divided into interrogation windows: for each window located at position ${{\boldsymbol{x}}}$, the local cell displacement ${\boldsymbol{\Delta }}{{\boldsymbol{r}}}$ is quantified by cross-correlating the intensity of two region-of-interest (ROI) images separated by ${{\Delta }}{{t}}$, which allows estimating the local velocity as ${\boldsymbol{v}}_{t}\left({\boldsymbol{x}}\right)=\frac{\boldsymbol{\Delta }{\boldsymbol{r}}}{{\Delta }{t}}$, where the index ${{t}}$ corresponds to the time of the frame pair used to compute the velocity field. We used ROIs of size $80\times 80$ px^2^, which are slightly larger than the typically observed cell size of $\sim 50$ px, with a spatial overlap factor of 50% between different ROIs. To improve statistics, we also performed a temporal average of the so-obtained velocity fields over chunks of 20 frames (200 min), again with a temporal overlap of 50%. The previous parameters were carefully optimized to find the best trade-off between increasing the spatiotemporal resolution and averaging a sufficient number of data samples to obtain smoother velocity maps, which will be indicated in the following with ${{{\boldsymbol{v}}}}_{{{t}}}\left({{\boldsymbol{x}}}\right)$. We then followed Garcia et al.^54^ to compute the total r.m.s. velocity as ${\boldsymbol{v}}_{\rm{RMS}}^{\rm{tot}}(t)={\sqrt{\left\langle |{\boldsymbol{v}}_{t}{({\boldsymbol{x}})}^{2}\right\rangle }}_{\boldsymbol{x}}$ and the drift-corrected r.m.s. velocity ${\boldsymbol{v}}_{\rm{RMS}}^{\rm{d.}{\rm{c}}.}\left(t\right)=\sqrt{{\left\langle |{\boldsymbol{v}}_{t}^{\rm{d}.{\rm{c}}.}({\boldsymbol{x}}){|}^{{{2}}}\right\rangle }_{\boldsymbol{x}}}$ as spatial averages of the velocity fields, where we have introduced the drift-collected velocity ${\boldsymbol{v}}_{t}^{{\rm{d}}{{.}}{\rm{c}}.}({\boldsymbol{x}})={\boldsymbol{v}}_{{{t}}}({\boldsymbol{x}})-{\left\langle {{\boldsymbol{v}}}_{{{t}}}({\boldsymbol{x}})\right\rangle }_{\boldsymbol{x}}$. In cell lines with no strong collective motion, ${\boldsymbol{v}}_{t}({\boldsymbol{x}})$ and ${\boldsymbol{v}}_{t}^{\mathrm{d.c.}}({\boldsymbol{x}})$ are similar, but in the presence of collective motion, these two quantities can differ substantially. As suggested by Garcia et al.^54^, we used the drift-corrected velocity to calculate the radial velocity–velocity correlation function, obtained as

$${C}_{\mathrm{vv}}(\delta {{x}},t)=\frac{\langle {\bf{v}}_{t}^{\rm{d.c.}}({\bf{x}}+{\delta {\bf{x}}, t})\cdot {\bf{v}}_{t}^{\rm{d.c.}}({\bf{x}},t)\rangle\!{\atop\bf{x}}}{\langle {{\bf{v}}_{t}^{\rm{d.c.}}({\bf{x}},t)}^{2}\rangle\!{\atop\bf{x}}}.$$

Furthermore, we fitted this function to a model exponential ${{\rm{e}}}^{\frac{{{\delta }}{\bf{x}}}{{{\xi }}}}$ to extract the spatial correlation length ${{\xi }}$ of the velocity field, quantifying the size of regions with similar velocities once the average monolayer velocity has been removed. Finally, to better visualize spatial correlations in the velocity field, we followed Malinverno et al.^55^ and calculated the alignment index ${{{a}}}_{{{t}}}\left({{\bf{x}}}\,\right)$ as the cosine of the angle between the average velocity vector of a single velocity field with every other velocity vector.

### Cell-stretching assay

Stretch chambers (STB-CH-4W, STREX cell-stretching systems) were autoclaved and coated with 10 μg ml^−1^ of collagen I (C8919, Sigma) and 10 μg ml^−1^ of fibronectin (341631, Sigma) diluted in PBS at 37 °C for 2 h. The coated chambers were washed three times with PBS before seeding 200,000 cells per well in the culture medium (88,888 cells cm^−^^2^). Cells were stretched the following day with a STREX cell-stretching system (model number STB-140-10) with 20% stretch (6.40 mm), 1-Hz frequency for 30 min and 1 h.

### Cell vibration assay

Flexible-bottomed silicone elastomer plates (BF-3001U, BioFlex) were coated with 10 μg ml^−1^ of collagen I (C8919, Sigma) and 10 μg ml^−1^ of fibronectin (341631, Sigma) diluted in PBS for 2 h at 37 °C. The coated chambers were washed three times with PBS before seeding 500,000–900,000 cells in the culture medium (52,083–93,759 cells cm^−^^2^). On the following day, stimulation sound files were played for 30 min and 6 h, 1 min off/1 min on in a frequency range of 50–250 Hz with a phonomimetic bioreactor^92^ connected to a Crown XLS 1502 amplifier.

### 3D spheroid assay

Spheroid formation in a 3D environment was assessed by embedding cells between two layers of Matrigel (Corning, 354230). First, the bottom of an angiogenesis 96-well µ-plate (89646, ibidi) was coated with 10 µl of 50% Matrigel diluted in the culture medium and centrifuged at 4 °C, 200*g* for 20 min followed by 1-h incubation at 37 °C. Next, wells were filled with 20 µl of cell suspension in 25% Matrigel diluted in the culture medium (500 cells per well), centrifuged for 10 min at 100*g* and incubated at 37 °C for 4 h. Wells were filled with the culture medium supplemented with 10 µg ml^−1^ function blocking antibodies or IgG control; mouse anti-IgG (31903, Invitrogen), mouse anti-human α3 integrin (P1B5, in-house hybridoma), mouse anti-human α6 integrin (P5G10, in-house hybridoma) and rat anti-human β1 integrin (mAb13, in-house hybridoma). Spheroid formation was imaged for 10 days with IncuCyte S3 Live-Cell Analysis system (×10 objective). The culture medium was changed every 2–3 days. Analysis was performed using OrganoSeg software^93^ and ImageJ (v1.54p).

### Wetting assay

Cells were seeded in a low-attachment round-bottom 96-well plate to allow the formation of spheroids. The following day, spheroids were transferred to a multiwell plate previously coated with 10 µg ml^−1^ of fibronectin (diluted in PBS, incubated overnight at 4 °C and washed twice with PBS). Spheroids were monitored as they wet the substrate by time-lapse imaging for 48 h using IXplore Live Microscope (Olympus Evident; ×4 objective, 10-min time frame). Analysis of spreading area over time was performed using ImageJ. The data were normalized to the area of the spheroid at time 0. To evaluate the impact of integrin perturbations, spheroids were treated with the blocking antibodies described above before starting the wetting experiment.

### Immunostaining

Coated (collagen I and fibronectin, as previously mentioned) µ-slide eight-well chambered coverslips (ibidi), standard culture plates (Corning) or Softwell Easy Coat (Matrigen) were fixed at the indicated endpoint with 4% paraformaldehyde in the culture medium for 10 min at RT. Cells were washed with PBS three times for 5 min. Permeabilization and blocking were performed using 0.3% Triton X-100 in 10% normal horse serum diluted in PBS for 20 min at RT. Cells were stained with primary antibodies diluted in 10% normal horse serum overnight at 4 °C. Cells were washed three times for 5 min with PBS and incubated with secondary antibodies diluted in PBS for 1 h at RT, followed by three 5-min washes with PBS. Samples were either imaged right away or stored at 4 °C covered from light until imaging.

The list of antibodies is provided in Supplementary Table 1.

### Imaging

Confocal imaging was performed with a 3i spinning-disc confocal (Marianas spinning-disc imaging system with a Yokogawa CSU-W1 scanning unit on an inverted Carl ZEISS Axio Observer Z1 microscope, Intelligent Imaging Innovations) with ×10 ZEISS Plan-Apochromat objective (without immersion, 2-mm working distance, 0.45 numerical aperture), ×40 ZEISS LD C-Apochromat objective (water immersion, 0.62-mm working distance, 1.1 numerical aperture) and ×63 ZEISS Plan-Apochromat objective (oil immersion, 0.19-mm working distance, 1.4 numerical aperture). Wide-field imaging was performed with Nikon Eclipse Ti2-E (Hamamatsu sCMOS Orca Flash4.0, Lumencor Spectra X LED excitation). Live imaging was performed with Incucyte S3 or ZOOM Live-Cell Analysis system.

### Confocal microscopy image analysis

FA count and size were assessed by the segmentation of FAs from the maximum intensity projections (MIPs) of a confocal microscopy image *z* stack (ten bottom slices; vinculin, ITGB1 and ILK staining) using ImageJ software (v1.54p). Junctional intensities were assessed by measuring the integrated density value in MIPs divided by the cell number in each FoV of the confocal microscopy images (β-catenin staining) using ImageJ software. Junction morphology was manually assessed by counting each junction type (linear, reticular and zipper like) per FoV in MIPs of the confocal microscopy images (β-catenin staining) using ImageJ software. Changes in the orientation (coherency) were assessed using the OrientationJ package in ImageJ software from MIPs of the confocal microscopy images (actin and β-catenin staining)^94^. The total intensities were assessed by measuring the integrated density value divided by total cell number in FoV in MIPs of the confocal microscopy images (β-catenin, E-cadherin, YAP and AMOTL2) using ImageJ software. Total/nuclear intensities were assessed by the segmentation of total cell (cytoplasm + nucleus) and nuclear areas based on actin and DAPI staining, and measuring the integrated density value in MIPs of the confocal microscopy images (β-catenin, YAP and AMOTL2 staining) using ImageJ software.

### Mass cytometry

Cells were grown on a 10-cm plate to 90% confluence, washed once with PBS and detached with cell dissociation buffer (number 13150-016, Gibco). The detached cells were dispensed into 15-ml Falcon tubes, centrifuged at 300*g* for 5 min followed by the removal of supernatant and mixing the pellet by pipetting. Cells were resuspended in 1 ml of serum-free medium. Then, 1 ml of 1-µM cisplatin in a serum-free medium was added to the cells for 5 min, mixed well by pipetting and incubated for 5 min at room temperature. The mixture was quenched with Cell Staining Buffer (Maxpar), 5× volume of the stained cells. Cells were centrifuged at 300*g* for 5 min, the supernatant was aspirated and the cells were resuspended by pipetting. Cells were washed with 4 ml of Cell Staining Buffer (Maxpar). Cells were counted and three million cells aliquoted into 5 ml of polypropylene tube followed by centrifugation at 300*g* for 5 min. The supernatant was aspirated and the cells were gently mixed by pipetting. Cells were resuspended in 50 µl of Cell Staining Buffer (Maxpar). Cells were then stained with the antibody panel (Supplementary Table 2), starting with Fc-blocking. Fc Receptor Blocking Solution was added 1:100 to each tube and incubated for 10 min at room temperature. 50 µl of the prepared antibody cocktail was added to each tube and gently mixed by pipetting and incubated at room temperature for 15 min. Samples were gently vortexed and incubated for an additional 15 min at room temperature. After a total of 30-min incubation, samples were washed by adding 2 ml of Cell Staining Buffer (Maxpar) to each tube, centrifuged at 300*g* for 5 min and the supernatant was removed. Sample wash was repeated three times and the cells were resuspended in residual volume by gently vortexing after final wash and aspiration. Cells were fixed with 1 ml of 1.6% formaldehyde diluted in PBS and gently vortexed before 10 min of incubation at room temperature. Samples were centrifuged at 800*g* for 5 min and the supernatant was removed. Samples were gently vortexed to resuspend in the residual volume. After cell staining, 1 ml of cell intercalation solution was prepared for each sample by diluting Cell-ID Intercalator-103Rh 1:1,000 into Fix and Perm Buffer (Maxpar) and mixed by vortexing. Then, 1 ml of intercalation solution was added to each tube and gently vortexed. Samples were incubated for 1 h at room temperature or left overnight at 4 °C (up to 48 h). Before acquisition with Helios (WB Injector), cells were centrifuged at 800*g* for 5 min and washed by adding 2 ml of Cell Staining Buffer (Maxpar), followed by another round of centrifugation. The supernatant was removed and the samples were gently vortexed to resuspend cells in the residual volume. Cells were washed by adding 2 ml of Cell Acquisition Solution (CAS; Maxpar) to each tube and gently vortexed before counting and transferring one million cells into a new tube. Tubes were centrifuged at 800*g* for 5 min, followed by a careful aspiration of the supernatant. Cells were gently vortexed to resuspend in the residual volume, and finally, one million cells were resuspended in 900-µl CAS. Cells were filtered into cell strainer cap tubes. Sufficient volume of 0.1× EQ beads to resuspend all the samples in the experiment were prepared by diluting one-part beads to nine-parts CAS. Cells were left pelleted until ready to run on Helios. Immediately before data acquisition, the cell concentration was adjusted to 1.0 × 10^6^ cells ml^−1^ and diluted by the EQ bead solution. Cells were filtered into cap tubes. Samples were run and the data were acquired with Helios CyTOF. Mass cytometry antibodies were either purchased from Fluidigm or self-conjugated.

### RNA sequencing

RNA was isolated from three biological replicates of cells (900,000 cells per well in a six-well plate; 93,750 cells cm^−^^2^) seeded on coated (collagen I and fibronectin) BioFlex plates. Cells were washed with cold PBS followed by RNA extraction using a NucleoSpin RNA kit (number 740955.250, Macherey-Nagel) as per the manufacturer’s instructions. The total RNA concentration was measured with Nanodrop and the samples were normalized by diluting with RNAse-free water. The sample quality was verified using Agilent Bioanalyser 2100, and the final concentrations were measured using Qubit/Quant-IT Fluorometric Quantitation (Life Technologies). Illumina Stranded Total RNA prep library was prepared using 100 ng of RNA as per the manufacturer’s instructions (Illumina Stranded mRNA Preparation and Ligation kit (Illumina) and sequenced with Novaseq 6000 (S4 instrument, v1.5 (Illumina), 2 × 50 bp, SP flow cell, two lanes (650–800M reads))). The library quality was verified using an Advanced Analytical Fragment Analyser. The sequencing data read quality was ensured using the FastQ (v0.11.14) and MultiQC (v1.5) tools^95^. Differentially expressed genes were assessed using Bioconductor R package ROTS (v1.14.0), defining genes with FDR < 0.05 as differentially expressed.

### Multiplexed fluorescence immunohistochemical staining and imaging

Multiplexed fluorescence immunohistochemical staining and imaging was performed in three cycles, as previously described^96^, for two sets of seven to eight antibodies and the nuclear marker DAPI (Supplementary Table 1), stained on two serial TMA sections. After the first round of staining and whole-slide imaging of the TMAs, the fluorescence signal was bleached, and the antibodies from the first round of staining were denatured, after which the second round of staining was performed. The process was repeated for the third round of staining. Imaging was performed using a ZEISS Axio Scan.Z1 slide scanner, with each round of staining recorded as an independent .czi image file containing up to five fluorescence channels.

### Image analysis of multiplexed TMA datasets

Images of individual TMA cores were extracted from the whole-slide images using the TMA dearrayer functionality in QuPath^97^. Images from the three staining rounds were registered using an affine image registration method operating through the pyStackReg Python dependency^98^, aligning the DAPI channels of the three staining rounds. Autofluorescence signal from red blood cells and other histology artefacts (for example, wrinkled or folded tissue section areas) were removed using a pixel classifier in ilastik^99^. Nuclei were segmented from the DAPI channel using a trained StarDist model^100^. The nuclear ROIs were expanded by 6 px to generate extranuclear ROIs. Pan-epithelial staining was used to threshold cells into epithelial and stromal compartments. A custom Python script^101^ was then used to calculate the fluorescence intensity in all channels for the relevant nuclear or extranuclear ROI in the relevant tissue compartments. Finally, patient-level average expression values were calculated for all cells and all TMA cores originating from the same patient. Five samples in which fewer than 100 cells could be quantified within the stromal or epithelial tissue compartment were excluded from further analyses to ensure a representative quantification of cellular phenotypes across the tumour tissue. A total of 193 samples were included in the analyses.

### Calculation of ECM, YAP and β-catenin scores

For the ECM scores, the median patient-level expressions (intensities) of stromal fibronectin, collagen I, SMA, laminin and vinculin were determined across the full patient dataset. Next, each patient was assigned one point for each instance that the expression of each of the above markers was above the dataset median. The sum of all points was determined as that patient’s ECM score. YAP scores were determined in the same way, with patients being assigned into the YAP-high group if their mean nuclear YAP expression in the tumour epithelium fell above the dataset median. All other patients were assigned into the YAP-low group.

### Survival analysis

Kaplan–Meier analysis was used to compare the survival outcomes between patient groups with different phenotypic signatures, with a log-rank test used to measure statistical significance. *P* ≤ 0.05 was used as a cut-off for statistical significance.

### Statistics and reproducibility

GraphPad Prism (v9.3.1) was used for all statistical analyses. Outliers were identified with 0.1% ROTS and indicated in the Source data. Data distribution was determined with the Shapiro–Wilk normality test. Two-sample testing was performed using Student’s *t*-test (unpaired, two-tailed) with Welch’s correction (normally distributed data) or non-parametric Mann–Whitney *U*-test (non-normally distributed data). Multiple comparisons were performed using either one-way analysis of variance (ANOVA; normally distributed data) or Kruskal–Wallis (non-normally distributed data) followed by an appropriate post hoc test, as indicated in the figure legends. Data are presented as column graphs, dot plots (mean ± standard deviation (s.d.)) or box plots (defined in the legends). *P* values less than 0.05 were considered to be statistically significant. Exact *P* values are provided in the figures where possible. Otherwise, *P* values are available in the Source data for each figure.

No statistical methods were used to predetermine the sample sizes, but our sample sizes were based on previous reports^101–104^. Data were reproduced in three or more biological replicates, unless otherwise indicated in the figure legends. Excluded data have been indicated in the Methods and figure legends. These pertain to the TMA samples in which five patient samples with fewer than 100 cells within the stromal or epithelial tissue compartment were excluded from further analyses to ensure a representative quantification of cellular phenotypes across tumour tissue, bringing the total samples analysed from 198 to 193. In addition, five patient samples with no available tumour staging information were excluded from analyses requiring a defined tumour stage, bringing the total samples analysed in these cases from 193 to 188.

The experiments were not randomized. However, animals were randomly assigned to cages (equal number of animals per cage) by the animal facility staff. Cages were chosen at random for experimentation. Mice assigned to different experimental conditions were run in parallel, and all animals were maintained under the same condition and were at the same developmental stage.

Experiments were not performed in a blinded fashion. Analysis software/statistical packages were used as detailed in the Methods for robust data analysis, removing user bias. In addition, appropriate controls were included in experiments and control versus treated samples were analysed in the same fashion.

### Reporting summary

Further information on research design is available in the Nature Portfolio Reporting Summary linked to this article.

## Online content

Any methods, additional references, Nature Portfolio reporting summaries, source data, extended data, supplementary information, acknowledgements, peer review information; details of author contributions and competing interests; and statements of data and code availability are available at 10.1038/s41563-025-02473-7.

## Supplementary information

Supplementary Information Supplementary Tables 1 and 2.

Reporting Summary

Supplementary Video 1 T1 VFC cell proliferation (single cells) on a 0.5-kPa-collagen- and fibronectin-coated hydrogel. Imaged using Incucyte (ZOOM) every 2 h for 116 h, ×10 magnification.

Supplementary Video 2 T3 VFC cell proliferation (single cells) on a 0.5-kPa-collagen- and fibronectin-coated hydrogel. Imaged using Incucyte (ZOOM) every 2 h for 116 h, ×10 magnification.

Supplementary Video 3 T1 VFC cell proliferation (single cells) on a 50-kPa-collagen- and fibronectin-coated hydrogel. Imaged using Incucyte (ZOOM) every 2 h for 116 h, ×10 magnification.

Supplementary Video 4 T3 VFC cell proliferation (single cells) on a 50-kPa-collagen- and fibronectin-coated hydrogel. Imaged using Incucyte (ZOOM) every 2 h for 116 h, ×10 magnification.

Supplementary Video 5 T1 VFC cell proliferation (single cells) on collagen- and fibronectin-coated plastic. Imaged using Incucyte (ZOOM) every 2 h for 116 h, ×10 magnification.

Supplementary Video 6 T3 VFC cell proliferation (single cells) on collagen- and fibronectin-coated plastic. Imaged using Incucyte (ZOOM) every 2 h for 116 h, ×10 magnification.

Supplementary Video 7 T1 VFC cell proliferation (single cells) on a 0.5-kPa Matrigel-coated hydrogel. Imaged using Incucyte (ZOOM) every 2 h for 116 h, ×10 magnification.

Supplementary Video 8 T3 VFC cell proliferation (single cells) on a 0.5-kPa Matrigel-coated hydrogel. Imaged using Incucyte (ZOOM) every 2 h for 116 h, ×10 magnification.

Supplementary Video 9 T1 VFC cell proliferation (single cells) on a 50-kPa Matrigel-coated hydrogel. Imaged using Incucyte (ZOOM) every 2 h for 116 h, ×10 magnification.

Supplementary Video 10 T3 VFC cell proliferation (single cells) on a 50-kPa Matrigel-coated hydrogel. Imaged using Incucyte (ZOOM) every 2 h for 116 h, ×10 magnification.

Supplementary Video 11 T1 VFC cell proliferation (single cells) on Matrigel-coated plastic. Imaged using Incucyte (ZOOM) every 2 h 116 h, ×10 magnification.

Supplementary Video 12 T3 VFC cell proliferation (single cells) on Matrigel-coated plastic. Imaged using Incucyte (ZOOM) every 2 h for 116 h, ×10 magnification.

Supplementary Video 13 NC cell proliferation (colony) on collagen- and fibronectin-coated plastic. Growth medium supplemented with anti-α3α6 antibody (P1B5 and P5G10, 10 µg ml^−1^) at 17 h. Imaged using Incucyte (S3) every 60 min for 23 h, ×20 magnification.

Supplementary Video 14 T1 VFC cell proliferation (colony) on collagen- and fibronectin-coated plastic. Growth medium supplemented with anti-α3α6 antibody (P1B5 and P5G10, 10 µg ml^−1^) at 17 h. Imaged using Incucyte (S3) every 60 min for 23 h, ×20 magnification.

Supplementary Video 15 T3 VFC cell proliferation (colony) on collagen- and fibronectin-coated plastic. Growth medium supplemented with anti-α3α6 antibody (P1B5 and P5G10, 10 µg ml^−1^) at 17 h. Imaged using Incucyte (S3) every 60 min for 23 h, ×20 magnification.

Supplementary Video 16 NC cell proliferation (colony) on collagen- and fibronectin-coated plastic. Growth medium supplemented with anti-E-cadherin antibody (DECMA-1) at 17 h. Imaged using Incucyte (S3) every 60 min for 23 h, ×20 magnification.

Supplementary Video 17 T1 VFC cell proliferation (colony) on collagen- and fibronectin-coated plastic. Growth medium supplemented with anti-E-cadherin antibody (DECMA-1) at 17 h. Imaged using Incucyte (S3) every 60 min for 23 h, ×20 magnification.

Supplementary Video 18 T3 VFC cell proliferation (colony) on collagen- and fibronectin-coated plastic. Growth medium supplemented with anti-E-cadherin antibody (DECMA-1) at 17 h. Imaged using Incucyte (S3) every 60 min for 23 h, ×20 magnification.

Supplementary Video 19 NC cell motility (monolayer) on collagen- and fibronectin-coated plastic. Growth medium supplemented with anti-IgG antibody (10 µg ml^−1^). Imaged using Incucyte (S3) every 10 min for 24 h, ×20 magnification.

Supplementary Video 20 NC cell motility (monolayer) on collagen- and fibronectin-coated plastic. Growth medium supplemented with anti-α3 integrin antibody (P1B5, 10 µgml^−1^). Imaged using Incucyte (S3) every 10 min for 24 h, ×20 magnification.

Supplementary Video 21 NC cell motility (monolayer) on collagen- and fibronectin-coated plastic. Growth medium supplemented with anti-α6 integrin antibody (P5G10, 10 µg ml^−1^). Imaged using Incucyte (S3) every 10 min for 24 h, ×20 magnification.

Supplementary Video 22 NC cell motility (monolayer) on collagen- and fibronectin-coated plastic. Growth medium supplemented with anti-α3α6 integrin antibody (P1B5 and P5G10, 10 µg ml^−1^). Imaged using Incucyte (S3) every 10 min for 24 h, ×20 magnification.

Supplementary Video 23 NC cell motility (monolayer) on collagen- and fibronectin-coated plastic. Growth medium supplemented with anti-β1 integrin antibody (mAb13, 10 µg ml^−1^). Imaged using Incucyte (S3) every 10 min for 24 h, ×20 magnification.

Supplementary Video 24 T1 VFC cell motility (monolayer) on collagen- and fibronectin-coated plastic. Growth medium supplemented with anti-IgG antibody (10 µg ml^−1^). Imaged using Incucyte (S3) every 10 min for 24 h, ×20 magnification.

Supplementary Video 25 T1 VFC cell motility (monolayer) on collagen- and fibronectin-coated plastic. Growth medium supplemented with anti-α3 integrin antibody (P1B5, 10 µg ml^−1^). Imaged using Incucyte (S3) every 10 min for 24 h, ×20 magnification.

Supplementary Video 26 T1 VFC cell motility (monolayer) on collagen- and fibronectin-coated plastic. Growth medium supplemented with anti-α6 integrin antibody (P5G10, 10 µg ml^−1^). Imaged using Incucyte (S3) every 10 min for 24 h, ×20 magnification.

Supplementary Video 27 T1 VFC cell motility (monolayer) on collagen- and fibronectin-coated plastic. Growth medium supplemented with anti-α3α6 integrin antibody (P1B5 and P5G10, 10 µg ml^−1^). Imaged using Incucyte (S3) every 10 min for 24 h, ×20 magnification.

Supplementary Video 28 T1 VFC cell motility (monolayer) on collagen- and fibronectin-coated plastic. Growth medium supplemented with anti-β1 integrin antibody (mAb13, 10 µg ml^−1^). Imaged using Incucyte (S3) every 10 min for 24 h, ×20 magnification.

Supplementary Video 29 T3 VFC cell motility (monolayer) on collagen- and fibronectin-coated plastic. Growth medium supplemented with anti-IgG antibody (10 µg ml^−1^). Imaged using Incucyte (S3) every 10 min for 24 h, ×20 magnification.

Supplementary Video 30 T3 VFC cell motility (monolayer) on collagen- and fibronectin-coated plastic. Growth medium supplemented with anti-α3 integrin antibody (P1B5, 10 µg ml^−1^). Imaged using Incucyte (S3) every 10 min for 24 h, ×20 magnification.

Supplementary Video 31 T3 VFC cell motility (monolayer) on collagen- and fibronectin-coated plastic. Growth medium supplemented with anti-α6 integrin antibody (P5G10, 10 µg ml^−1^). Imaged using Incucyte (S3) every 10 min for 24 h, ×20 magnification.

Supplementary Video 32 T3 VFC cell motility (monolayer) on collagen- and fibronectin-coated plastic. Growth medium supplemented with anti-α3α6 integrin antibody (P1B5 and P5G10, 10 µg ml^−1^). Imaged using Incucyte (S3) every 10 min for 24 h, ×20 magnification.

Supplementary Video 33 T3 VFC cell motility (monolayer) on collagen- and fibronectin-coated plastic. Growth medium supplemented with anti-β1 integrin antibody (mAb13, 10 µg ml^−1^). Imaged using Incucyte (S3) every 10 min for 24 h, ×20 magnification.

Supplementary Video 34 Representative phase contrast videos of NC and VFC T1 and T3 spheroids wetting the surface. Frame rate: 1 frame every 10 min for a total of 48 h.

## Source data

Source Data Fig. 1 Statistical source data.

Source Data Fig. 2 Statistical source data.

Source Data Fig. 3 Statistical source data.

Source Data Fig. 4 Statistical source data.

Source Data Fig. 5 Statistical source data.

Source Data Fig. 5 Unprocessed western blots.

Source Data Fig. 6

Source Data Extended Data Fig. 1 Statistical source data.

Source Data Extended Data Fig. 1 Unprocessed western blots.

Source Data Extended Data Fig. 2 Statistical source data.

Source Data Extended Data Fig. 2 Unprocessed western blots.

Source Data Extended Data Fig. 3 Statistical source data.

Source Data Extended Data Fig. 4 Statistical source data.

Source Data Extended Data Fig. 4 Unprocessed western blots.

Source Data Extended Data Fig. 5 Statistical source data.

Source Data Extended Data Fig. 6 Statistical source data.

Source Data Extended Data Fig. 7 Statistical source data.

Source Data Extended Data Fig. 8 Statistical source data.

Source Data Extended Data Fig. 9 Statistical source data.

## Data Availability

The RNA-sequencing data have been deposited at Gene Expression Omnibus (GEO) and are publicly available as of the date of publication (GEO accession number: GSE297099). Data supporting the findings of this study are available within the Article and its Supplementary Information. Statistical source data and uncropped and unprocessed blots are provided for all the figures. The use of T1 and T3 cell lines for other purposes requires ethical approval and permission from the Auria Biobank. Source data are provided with this paper.

## Code availability

The custom Python script used in the image analysis of multiplexed TMA datasets was used in a previous publication^101^ and is publicly available via GitHub (https://github.com/WickstromLab/Punovuori-et-al.-Cell-2024) and Zenodo (10.5281/zenodo.17913603)^105^. The PIV algorithm is publicly available via GitHub (https://github.com/somexlab/cellpiv/) and Zenodo (10.5281/zenodo.17898956)^106^.
