# Supplementary material for: Restoring the tumour mechanophenotype of vocal fold cancer reverts its malignant properties
Source: Nat Mater. 2026 Feb 20;25(5):868–82. doi: 10.1038/s41563-025-02473-7 (PMC13143829; doi:10.1038/s41563-025-02473-7)
Supplement: Supplementary file 1 — Supplementary Tables 1 and 2. [file 41563_2025_2473_MOESM1_ESM.pdf]

nature materials

Supplementary information https://doi.org/10.1038/s41563-025-02473-7

Restoring the tumour mechanophenotype of vocal fold cancer reverts its malignant properties

In the format provided by the authors and unedited

Supplementary information

Supplementary tables

Supplementary Table 1: Details of the primary antibodies and reagents used in the study. IF: immunofluorescence, MP: multiplex fluorescence immunohistochemistry, WB: western blot.

Reagent	***Dilution***	***Application***	***Supplier***	***Catalog no.***
4',6-Diamidino-2-Phenylindole, Dihydrochloride (DAPI)	1:2000	IF	Life technologies	D1306
Sir-Actin	1: 1000	IF	Tebu-Bio	SC001
Mouse anti-active β1 (clone 12G10)	1:50	IF	In-house production from hybridoma	
Mouse anti-β1 integrin	1:1000	WB	BD Biosciences	610468
Mouse anti-α3 integrin (ASC-1)	1:100	IF	Abcam	ab228425
Rabbit anti-α3 integrin	1:1000	MP	Abcam	ab131055
Rabbit anti-α3 integrin	1:1000	WB	Abcam	ab131055
Mouse anti-β4 integrin	1:100	IF, WB	Millipore	MAB1964
Rat anti-β4 integrin	1:100	MP	Abcam	ab95583
Rat anti-α6 integrin (CD49f, cloneGoH3)	1:100	IF	Serotec	MCA699
Rat anti-α6 integrin	1:500	MP	Novus	85747
Rabbit anti-α6 integrin	1:1000	WB	Abcam	ab97760
Rabbit anti-β-catenin (E247)	1:100	IF	Abcam	ab32572
Mouse anti-β-catenin	1:500	MP	Cell Marque	224M-14
Mouse anti-CD151	1:100	IF	Abcam	ab33315
Rabbit anti-phospho-MLC 2 (Thr18/Ser19)	1:100, 1:1000, 1:1000	IF, MP, WB	Cell Signaling Technology	3674
Rabbit anti-COLXVII (EPR18614)	1:100, 1:500, 1:1000	IF, MP, WB	Abcam	ab184996
Mouse anti-vinculin	1:100, 1:1000	IF, WB	Sigma	V9131
Rat anti-Hsc70/Hsp73	1:1000	WB	Enzo	ADI-SPA-815
Guinea pig anti-keratin 14	1: 100, 1:1000	IF, WB	Covance	PRB-155P
Guinea pig anti-keratin 14	1: 1000	MP	Progen	GP-CK14
Mouse anti-pan cytokeratin	1:150	MP	Abcam	ab7753
Mouse anti-pan cytokeratin	1: 100	MP	Invitrogen	MA5 13156
Rabbit anti-Fibronectin	1:1000, 1:1000	MP, WB	Sigma	F3648
Rabbit anti-Collagen I	1:1000	MP	Novus	NB600-408
Rabbit anti-pan-laminin	1:100	MP	Sigma	L9393

Mouse anti-E-cadherin	1:200	MP	BD Biosciences	610182
Rabbit anti-E-cadherin	1:100, 1:1000	IF, WB	Cell Signaling Technology	3195
Mouse anti-α-SMA	1:2000	MP	DAKO	M0851
Rabbit anti-AMOTL2	1:100, 1:1000	IF, WB	Proteintech	23351-1-AP
Mouse anti-YAP	1:100, 1:50	IF, MP	Santa Cruz	sc-101199

Supplementary Table 2: The list of antibodies used for Mass Cytometry.

Metal tag	Target protein	Conjugation
106CD	α11 integrin	Self-conjugated
110CD	HER3	Self-conjugated
111CD	α3 integrin (CD49c)	Self-conjugated
112CD	EGFR	Self-conjugated
113CD	CD10	Self-conjugated
114CD	αv integrin (CD51)	Self-conjugated
116CD	HER4	Self-conjugated
89Y	αIIb integrin (CD41)	3089004B
141PR	EpCAM (CD326)	3141006B
142ND	PETA-3 (CD151)	3142011B
143ND	N-Cadherin (CD325)	3143016B
144ND	Syndecan-4	Self-conjugated
145ND	Syndecan-1 (CD138)	3145003B
146ND	β3 integrin (CD61)	3146011B
147SM	ALCAM (CD166)	Self-conjugated
148ND	HER2 (ErbB2/EGFR2)	3148011A
149SM	CD34	3149013B
150ND	αvβ3 integrin (CD51/61)	3150026B
151EU	ICAM-2 (CD102)	3151015B
152SM	αvβ5 integrin	Self-conjugated
153EU	β6 integrin	Self-conjugated
154SM	Notch1	Self-conjugated
155GD	α8 integrin	Self-conjugated
156GD	β1 integrin (CD29)	3156007B
158GD	E-Cadherin (CD324)	3158018B
159TB	LAT1 (CD98)	3159022B
160GD	α5 integrin (CD49e)	3160015B
161DY	α2 integrin (CD49b)	3161012B
162DY	β7 integrin	3162026B
163DY	α1 integrin (CD49a)	3163015B
164DY	α6 integrin (CD49F)	3164006B
165HO	Notch2	3165026B
166ER	CD44	3166001B
167ER	Notch3	Self-conjugated
168ER	α9β1 integrin	3168013B
169TM	CD24	3169004B
170ER	ICAM-1 (CD54)	3170014B
171YB	CD9	3171009B

172YB	Neuropilin-1 (CD304)	Self-conjugated
173YB	β4 integrin (CD104)	3173008B
174YB	α4 integrin (CD49d)	3174018B
175LU	β8 integrin	Self-conjugated
176YB	NCAM (CD56)	3176001B
209BI	CD47	3209004B
