# Supplementary material for: Restoring the tumour mechanophenotype of vocal fold cancer reverts its malignant properties
Source: Nat Mater. 2026 Feb 20;25(5):868–82. doi: 10.1038/s41563-025-02473-7 (PMC13143829; doi:10.1038/s41563-025-02473-7)
Supplement: Supplementary file 2 — Reporting Summary [file 41563_2025_2473_MOESM2_ESM.pdf]

nature portfolio

Corresponding author(s): Johanna Ivaska

Last updated by author(s): Nov 12, 2025

Reporting Summary

Nature Portfolio wishes to improve the reproducibility of the work that we publish. This form provides structure for consistency and transparency in reporting. For further information on Nature Portfolio policies, see our Editorial Policies and the Editorial Policy Checklist.

Statistics

For all statistical analyses, confirm that the following items are present in the figure legend, table legend, main text, or Methods section.

n/a	Confirmed	
☐	☒	The exact sample size (*n*) for each experimental group/condition, given as a discrete number and unit of measurement
☐	☒	A statement on whether measurements were taken from distinct samples or whether the same sample was measured repeatedly
☐	☒	The statistical test(s) used AND whether they are one- or two-sided<br>*Only common tests should be described solely by name; describe more complex techniques in the Methods section.*
☒	☐	A description of all covariates tested
☐	☒	A description of any assumptions or corrections, such as tests of normality and adjustment for multiple comparisons
☐	☒	A full description of the statistical parameters including central tendency (e.g. means) or other basic estimates (e.g. regression coefficient) AND variation (e.g. standard deviation) or associated estimates of uncertainty (e.g. confidence intervals)
☐	☒	For null hypothesis testing, the test statistic (e.g. *F*, *t*, *r*) with confidence intervals, effect sizes, degrees of freedom and *P* value noted<br>*Give P values as exact values whenever suitable.*
☒	☐	For Bayesian analysis, information on the choice of priors and Markov chain Monte Carlo settings
☒	☐	For hierarchical and complex designs, identification of the appropriate level for tests and full reporting of outcomes
☐	☒	Estimates of effect sizes (e.g. Cohen's *d*, Pearson's *r*), indicating how they were calculated

Our web collection on statistics for biologists contains articles on many of the points above.

Software and code

Policy information about availability of computer code

Data collection	Data was collected as indicated in the materials and methods. All instruments used were commercially available and controlled using software provided by the manufacturer, unless otherwise stated.
Data analysis	Data analyses were performed as described in the methods. Custom python scripts were used for analysis of TMA datasets and PIV analysis. A full code availability statement has been provided in the manuscript: "The custom python script used in the image analysis of multiplexed TMA datasets was used in a previous publication (REF 101) and is publicly available (https://github.com/WickstromLab/Punovuori-et-al.-Cell-2024). The PIV algorithm is also publicly available (https://github.com/somexlab/)."

For manuscripts utilizing custom algorithms or software that are central to the research but not yet described in published literature, software must be made available to editors and reviewers. We strongly encourage code deposition in a community repository (e.g. GitHub). See the Nature Portfolio guidelines for submitting code & software for further information.

Data

Policy information about availability of data

All manuscripts must include a data availability statement. This statement should provide the following information, where applicable:

- Accession codes, unique identifiers, or web links for publicly available datasets
- A description of any restrictions on data availability
- For clinical datasets or third party data, please ensure that the statement adheres to our policy

The RNA sequencing data have been deposited at Gene Expression Omnibus (GEO) and are publicly available as of the date of publication (GEO accession number: GSE297099). Data supporting the findings of this study are available within the paper and its source data supplementary information files. Statistical source data and uncropped and unprocessed blots are provided for all figures. The use of T1 and T3 cell lines for other purposes requires ethical approval and permission from the Auria Biobank.

Research involving human participants, their data, or biological material

Policy information about studies with human participants or human data. See also policy information about sex, gender (identity/presentation), and sexual orientation and race, ethnicity and racism.

Reporting on sex and gender	NA
Reporting on race, ethnicity, or other socially relevant groupings	NA
Population characteristics	NA
Recruitment	Patient samples were obtained at the Department of Otorhinolaryngology-Head and Neck Surgery at Turku University Hospital under the Finnish Biobank Act with written informed consent from the sample donors (§279, 9/2001).
Ethics oversight	Finnish Biobank Act (§279, 9/2001)

Note that full information on the approval of the study protocol must also be provided in the manuscript.

Field-specific reporting

Please select the one below that is the best fit for your research. If you are not sure, read the appropriate sections before making your selection.

☒ Life sciences ☐ Behavioural & social sciences ☐ Ecological, evolutionary & environmental sciences

For a reference copy of the document with all sections, see nature.com/documents/nr-reporting-summary-flat.pdf

Life sciences study design

All studies must disclose on these points even when the disclosure is negative.

Sample size	No statistical method was used to predetermine sample size but our sample sizes are based on previous reports including PMID: 39471809, 27488962, 37844244, 35677646. These have been indicated in the Methods statistics and reproducibility section.<br><br>Experiments were performed at least three times, unless otherwise stated. For imaging data, representative images are shown. For those experiments with < 3 independent replicates, sufficient sample sizes (cells, measurements, etc) were used to ensure results are scientifically relevant.
Data exclusions	Outliers were identified with 0.1 % ROTS and indicated in the source data.<br>Excluded data have been indicated in the methods and figure legends. These pertain to the TMA samples where five patient samples with fewer than 100 cells within the stromal or epithelial tissue compartment were excluded from further analyses to ensure representative quantification of cellular phenotypes across tumour tissue bringing the total samples analysed from 198 to 193. In addition, five patient samples with no available tumour staging information were excluded from analyses requiring a defined tumour stage bringing the total samples analysed in these cases from 193 to 188.
Replication	To ensure reproducibility, experiments were replicated at least three times (unless otherwise stated in the figure legends). Key experiments were performed at different times and with different batches of cells.
Randomization	The experiments were not randomized. However, animals were randomly assigned to cages (equal number of animals per cage) by the animal facility staff. Cages were chosen at random for experimentation. Mice assigned to different experimental conditions were run in parallel and all animals were maintained under the same condition and were at the same developmental stage. For imaging (immunofluorescence, Incucyte, atomic force microscopy), images were taken of multiple fields (or individual cells/spheroids) from the same sample at different locations.

Blinding	Experiments were not performed in a blinded fashion. Analysis software/statistical packages were used as detailed in the methods for robust data analysis, removing user bias. In addition, appropriate controls were included in experiments and control versus treated samples were analysed in the same fashion.

Reporting for specific materials, systems and methods

We require information from authors about some types of materials, experimental systems and methods used in many studies. Here, indicate whether each material, system or method listed is relevant to your study. If you are not sure if a list item applies to your research, read the appropriate section before selecting a response.

Materials & experimental systems

n/a	Involved in the study
☐	☒ Antibodies
☐	☒ Eukaryotic cell lines
☒	☐ Palaeontology and archaeology
☒	☐ Animals and other organisms
☒	☐ Clinical data
☒	☐ Dual use research of concern
☒	☐ Plants

Methods

n/a	Involved in the study
☒	☐ ChIP-seq
☒	☐ Flow cytometry
☒	☐ MRI-based neuroimaging

Antibodies

Antibodies used	All antibodies used are indicated in the Supplementary tables 1 and 2 in the Supplementary information file.<br>These include the following (antibody, dilution, application, supplier, cat. no.): Mouse anti-active β1 (clone 12G10), 1:50, IF, In-house production from hybridoma; Mouse anti-β1 integrin, 1:1000, WB, BD Biosciences, 610468; Mouse anti-α3 integrin (ASC-1), 1:100, IF, Abcam, ab228425; Rabbit anti-α3 integrin, 1:1000, MP, Abcam, ab131055; Rabbit anti-α3 integrin, 1:1000, WB, Abcam, ab131055; Mouse anti-β4 integrin, 1:100, IF, WB, Millipore, MAB1964; Rat anti-β4 integrin, 1:100, MP, Abcam, ab95583; Rat anti-α6 integrin (CD49f, cloneGoH3), 1:100, IF, Serotec, MCA699; Rat anti-α6 integrin, 1:500, MP, Novus, 85747; Rabbit anti-α6 integrin, 1:1000, WB, Abcam, ab97760; Rabbit anti-β-catenin (E247), 1:100, IF, Abcam, ab32572; Mouse anti-β-catenin, 1:500, MP, Cell Marque, 224M-14; Mouse anti-CD151, 1:100, IF, Abcam, ab33315; Rabbit anti-phospho-MLC 2 (Thr18/Ser19), 1:100, 1:1000, 1:1000, IF, MP, WB, Cell Signaling Technology, 3674; Rabbit anti-COLXVII (EPR18614), 1:100, 1:500, 1:1000, IF, MP, WB, Abcam, ab184996; Mouse anti-vinculin, 1:100, 1:1000, IF, WB, Sigma, V9131; Rat anti-Hsc70/Hsp73, 1:1000, WB, Enzo, ADI-SPA-815; Guinea pig anti-keratin 14, 1: 100, 1:1000, IF, WB, Covance, PRB-155P; Guinea pig anti-keratin 14, 1: 1000, MP, Progen, GP-CK14; Mouse anti-pan cytokeratin, 1:150, MP, Abcam, ab7753; Mouse anti-pan cytokeratin, 1: 100, MP, Invitrogen, MA5 13156; Rabbit anti-Fibronectin, 1:1000, 1:1000, MP, WB, Sigma, F3648; Rabbit anti-Collagen I, 1:1000, MP, Novus, NB600-408; Rabbit anti-pan-laminin, 1:100, MP, Sigma, L9393; Mouse anti-E-cadherin, 1:200, MP, BD Biosciences, 610182; Rabbit anti-E-cadherin, 1:100, 1:1000, IF, WB, Cell Signaling Technology, 3195; Mouse anti-α-SMA, 1:2000, MP, DAKO, M0851; Rabbit anti-AMOTL2, 1:100, 1:1000, IF, WB, Proteintech, 23351-1-AP; Mouse anti-YAP, 1:100, 1:50, IF, MP, Santa Cruz, sc-101199.<br><br>Antibodies used in mass cytof include: (metal tag, target, conjugation (either self conjugation or cat. no. of conjugated antibody)): 106CD, α11 integrin, Self-conjugated; 110CD, HER3, Self-conjugated; 111CD, α3 integrin (CD49c), Self-conjugated; 112CD, EGFR, Self-conjugated; 113CD, CD10, Self-conjugated; 114CD, αv integrin (CD51), Self-conjugated; 116CD, HER4 , Self-conjugated; 89Y, αIIb integrin (CD41), 3089004B; 141PR, EpCAM (CD326), 3141006B; 142ND, PETA-3 (CD151), 3142011B; 143ND, N-Cadherin (CD325), 3143016B; 144ND, Syndecan-4, Self-conjugated; 145ND, Syndecan-1 (CD138), 3145003B; 146ND, b3 integrin (CD61), 3146011B; 147SM, ALCAM (CD166) , Self-conjugated; 148ND, HER2 (ErbB2/EGFR2), 3148011A; 149SM, CD34, 3149013B; 150ND, avb3 integrin (CD51/61), 3150026B; 151EU, ICAM-2 (CD102), 3151015B; 152SM, avb5 integrin, Self-conjugated; 153EU, b6 integrin, Self-conjugated; 154SM, Notch1, Self-conjugated; 155GD, a8 integrin, Self-conjugated; 156GD, b1 integrin (CD29), 3156007B; 158GD, E-Cadherin (CD324), 3158018B; 159TB, LAT1 (CD98), 3159022B; 160GD, a5 integrin (CD49e), 3160015B; 161DY, a2 integrin (CD49b), 3161012B; 162DY, b7 integrin, 3162026B; 163DY, a1 integrin (CD49a), 3163015B; 164DY, a6 integrin (CD49F), 3164006B; 165HO, Notch2, 3165026B; 166ER, CD44, 3166001B; 167ER, Notch3, Self-conjugated; 168ER, a9b1 integrin, 3168013B; 169TM, CD24, 3169004B; 170ER, ICAM-1 (CD54), 3170014B; 171YB, CD9, 3171009B; 172YB, Neuropilin-1 (CD304), Self-conjugated; 173YB, b4 integrin (CD104), 3173008B; 174YB, a4 integrin (CD49d), 3174018B; 175LU, b8 integrin, Self-conjugated; 176YB, NCAM (CD56), 3176001B; 209BI, CD47, 3209004B.
Validation	Antibodies were not separately validated but have been used routinely by others and us. The manufacturer catalogue numbers are provided in the Supplementary Tables 1 and 2 in the Supplementary Information file. In addition, siRNA knockdown experiments included in the manuscript were used as a form of validation of the antibody.

Eukaryotic cell lines

Policy information about cell lines and Sex and Gender in Research

Cell line source(s)	UT-SCC-11 (T1) and UT-SCC-103 (T3) cell lines generated at Turku University Hospital have undergone scientific evaluation by Auria Biobank with a positive decision of release (AB22-7195) to be used in the study. Use of these cell lines for other purposes requires ethical approval and permission from the Auria Biobank.
Authentication	None of the cell lines were separately authenticated by the authors.

Mycoplasma contamination	Cell lines used in this study were regularly tested and confirmed negative for mycoplasma.
Commonly misidentified lines (See ICLAC register)	No commonly misidentified cell lines were used (ICLAC register version 13).

Plants

Seed stocks	N/A
Novel plant genotypes	N/A
Authentication	N/A
