# Supplementary material for: Restoring the tumour mechanophenotype of vocal fold cancer reverts its malignant properties
Source: Nat Mater. 2026 Feb 20;25(5):868–82. doi: 10.1038/s41563-025-02473-7 (PMC13143829; doi:10.1038/s41563-025-02473-7)
Supplement: Supplementary file 42 — Unprocessed western blots. [file 41563_2025_2473_MOESM42_ESM.pdf]

Fig. 5b and j

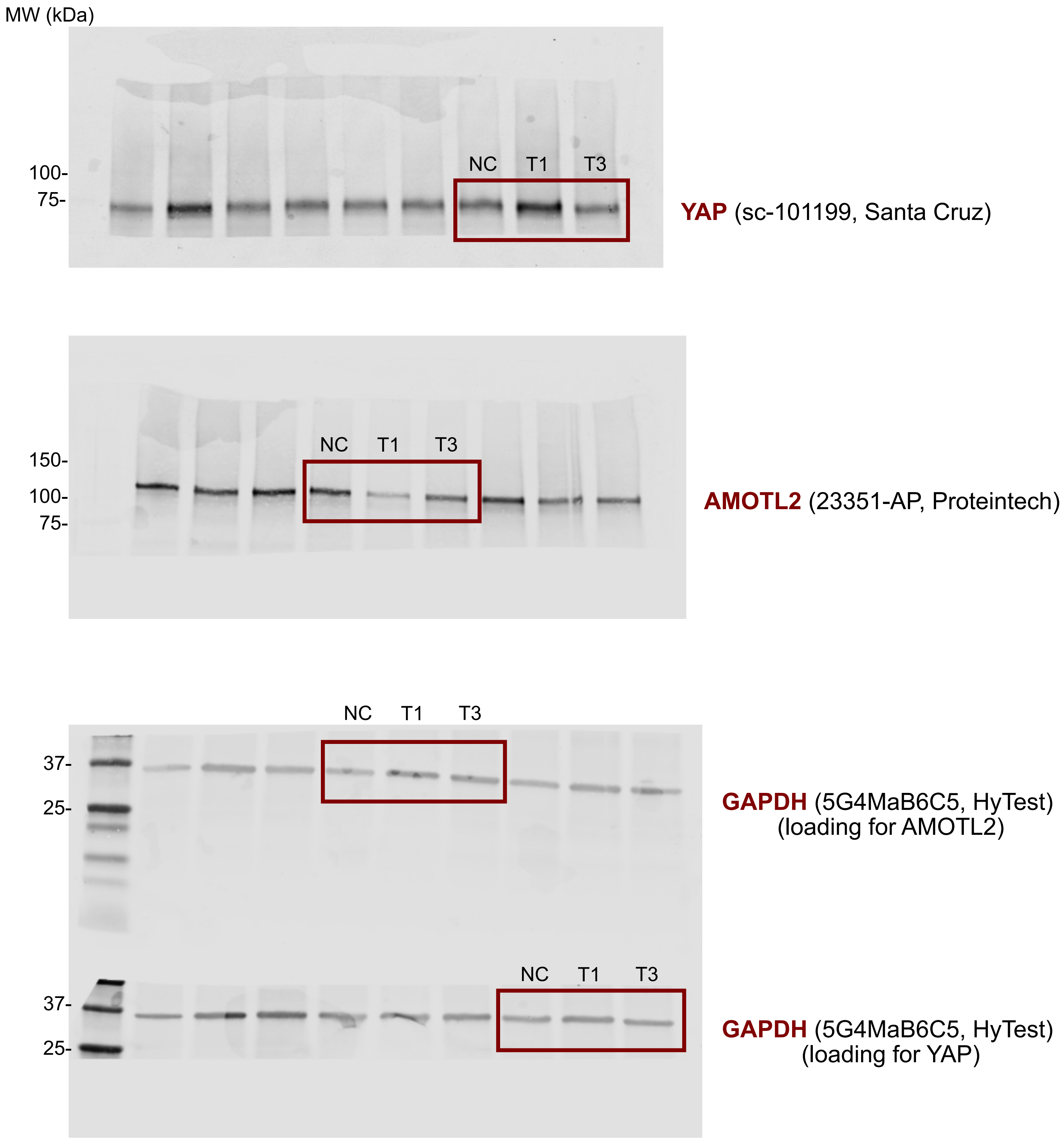
MW (kDa)
100-
75-
NC T1 T3
YAP (sc-101199, Santa Cruz)
150-
100-
75-
NC T1 T3
AMOTL2 (23351-AP, Proteintech)
NC T1 T3
37-
25-
GAPDH (5G4MaB6C5, HyTest)
(loading for AMOTL2)
NC T1 T3
37-
25-
GAPDH (5G4MaB6C5, HyTest)
(loading for YAP)
