# Supplementary figures and images for: Restoring the tumour mechanophenotype of vocal fold cancer reverts its malignant properties
Source: Nat Mater. 2026 Feb 20;25(5):868–82. doi: 10.1038/s41563-025-02473-7 (PMC13143829; doi:10.1038/s41563-025-02473-7)

Extended data Figure 1

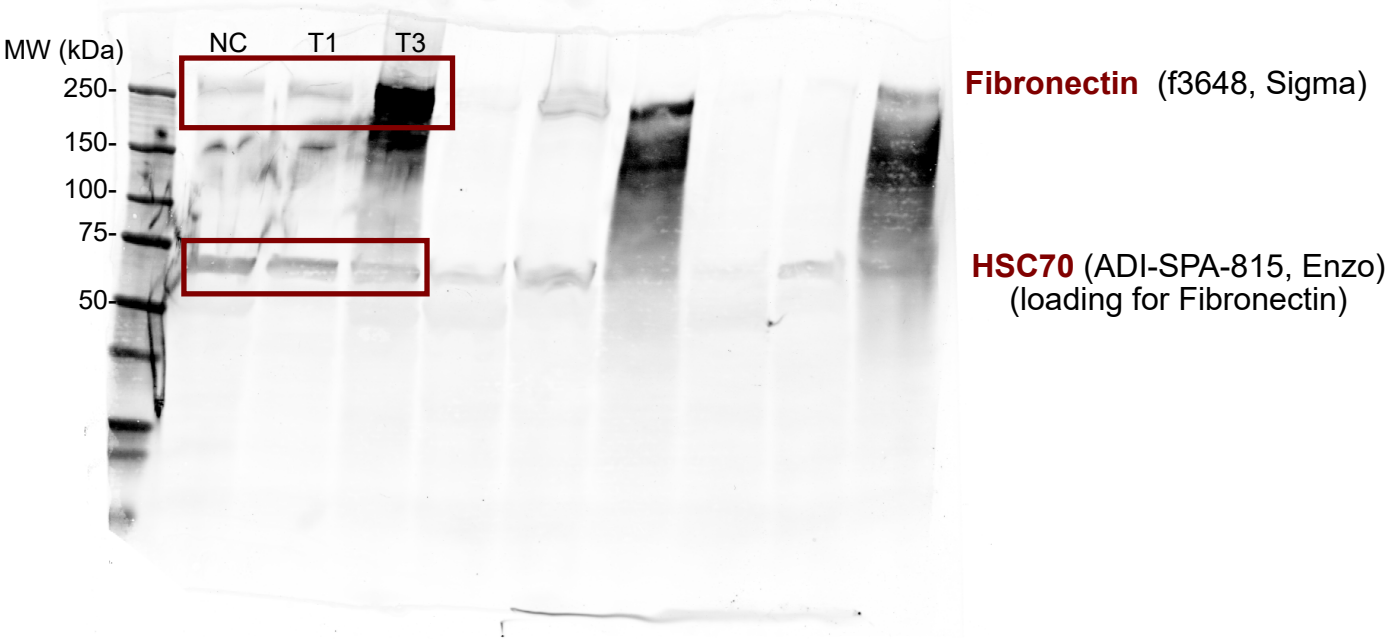

Supplement: Supplementary file 45 — Unprocessed western blots. [file 41563_2025_2473_MOESM45_ESM.pdf]

Extended data Figure 2d

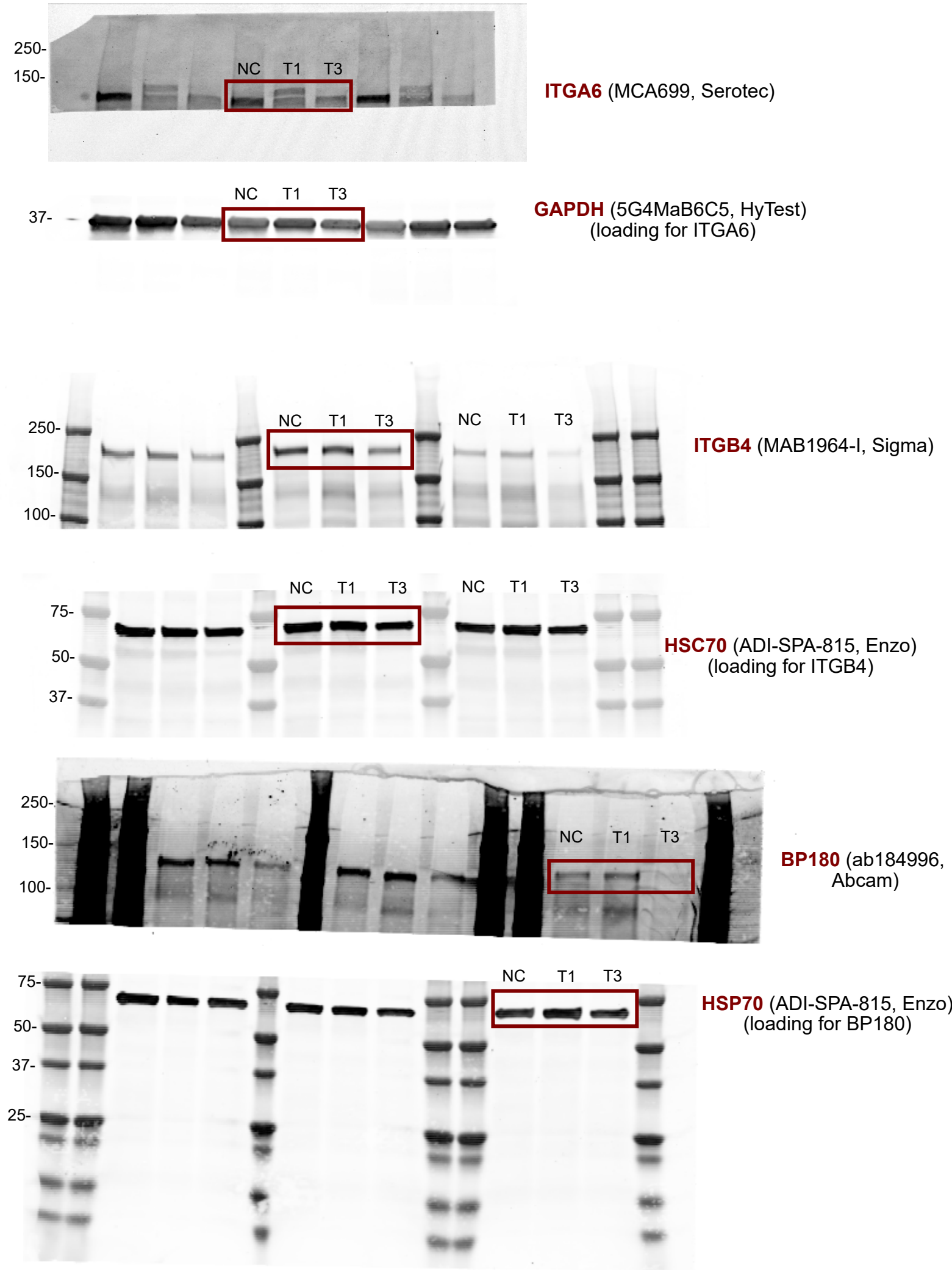

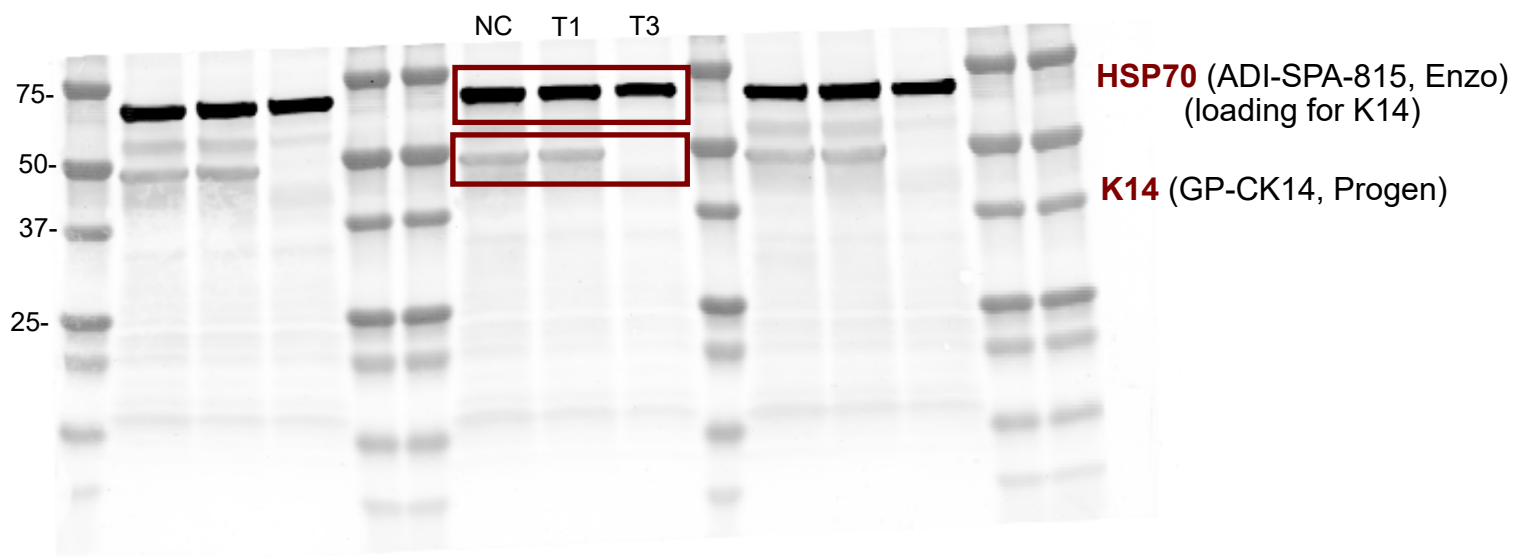

Supplement: Supplementary file 47 — Unprocessed western blots. [file 41563_2025_2473_MOESM47_ESM.pdf]

Extended data Figure 4a

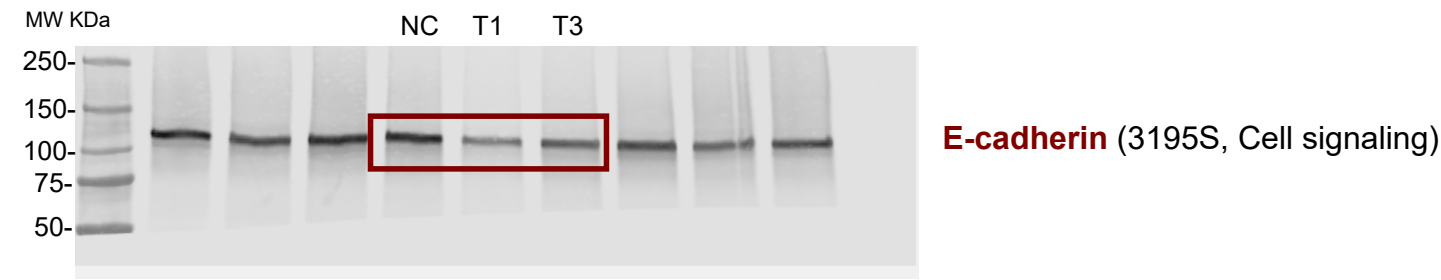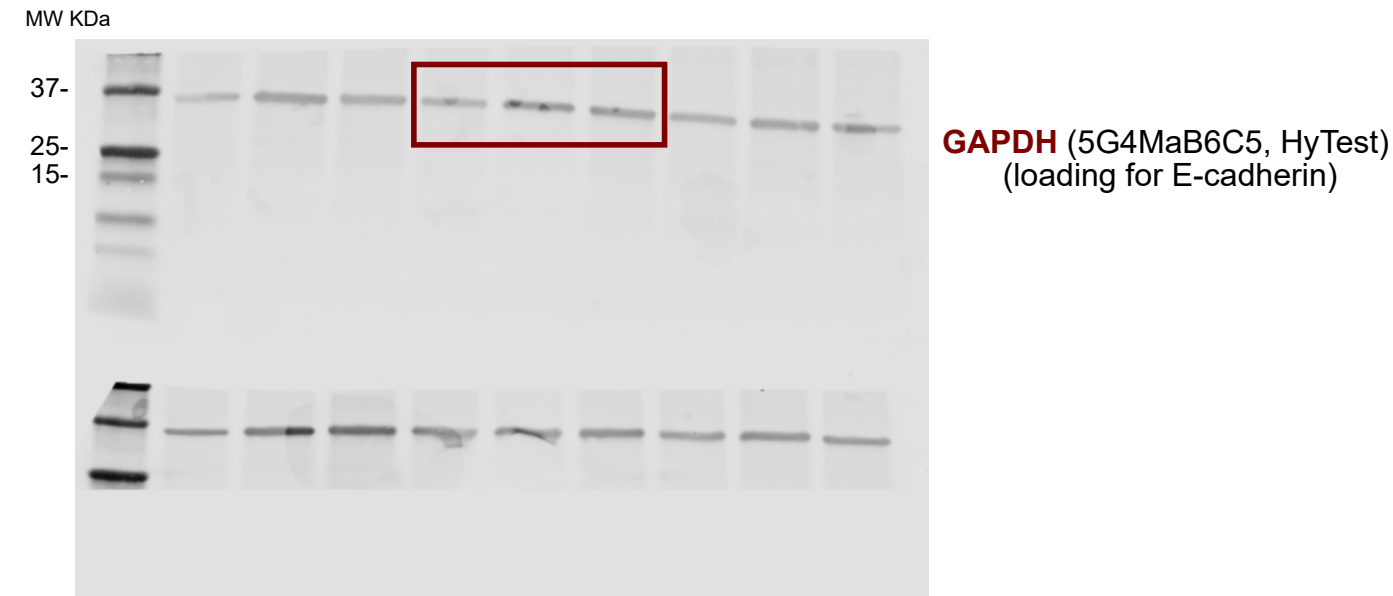

Supplement: Supplementary file 50 — Unprocessed western blots. [file 41563_2025_2473_MOESM50_ESM.pdf]
